# Anti-ferroptosis exosomes engineered for targeting M2 microglia to improve neurological function in ischemic stroke

**DOI:** 10.1186/s12951-024-02560-y

**Published:** 2024-05-27

**Authors:** Yong Wang, Zhuohang Liu, Luyu Li, Zengyu Zhang, Kai Zhang, Min Chu, Yang Liu, Xueyu Mao, Di Wu, Dongsheng Xu, Jing Zhao

**Affiliations:** 1 Department of Neurology, Minhang Hospital, Fudan University, Shanghai, 201100 China; 2 Department of Neurology, Zhongshan Hospital, Fudan University, Shanghai, 200030 China; 3 Department of Dermatology, Shanghai Ninth People’s Hospital, Shanghai Jiao Tong University School of Medicine, Shanghai, 200001 China; 4 Department of Cardiovascular Medicine, Pujiang Traditional Chinese Medicine Hospital, Zhejiang, 322200 China; 5 College of Rehabilitation Science, Shanghai University of Traditional Chinese Medicine, Shanghai, 200120 China; 6 Engineering Research Center of Traditional Chinese Medicine Intelligent Rehabilitation, Ministry of Education, Shanghai, 200120 China; 7 Institute of Healthy Yangtze River Delta, Shanghai Jiao Tong University, Shanghai, 200001 China

**Keywords:** Ischemic stroke, M2 microglia, Engineered exosomes, Inflammatory microenvironment, Lipid peroxidation, Ferroptosis susceptibility

## Abstract

**Background:**

Stroke is a devastating disease affecting populations worldwide and is the primary cause of long-term disability. The inflammatory storm plays a crucial role in the progression of stroke. In the acute phase of ischemic stroke, there is a transient increase in anti-inflammatory M2 microglia followed by a rapid decline. Due to the abundant phospholipid in brain tissue, lipid peroxidation is a notable characteristic of ischemia/reperfusion (I/R), constituting a structural foundation for ferroptosis in M2 microglia. Slowing down the decrease in M2 microglia numbers and controlling the inflammatory microenvironment holds significant potential for enhancing stroke recovery.

**Results:**

We found that the ferroptosis inhibitor can modulate inflammatory response in MCAO mice, characterizing that the level of M2 microglia-related cytokines was increased. We then confirmed that different subtypes of microglia exhibit distinct sensitivities to I/R-induced ferroptosis. Adipose-derived stem cells derived exosome (ADSC-Exo) effectively decreased the susceptibility of M2 microglia to ferroptosis via Fxr2/Atf3/Slc7a11, suppressing the inflammatory microenvironment and promoting neuronal survival. Furthermore, through plasmid engineering, a more efficient M2 microglia-targeted exosome, termed M2pep-ADSC-Exo, was developed. In vivo and in vitro experiments demonstrated that M2pep-ADSC-Exo exhibits significant targeting specificity for M2 microglia, further inhibiting M2 microglia ferroptosis and improving neurological function in ischemic stroke mice.

**Conclusion:**

Collectively, we illustrated a novel potential therapeutic mechanism that Fxr2 in ADSC-Exo could alleviate the M2 microglia ferroptosis via regulating Atf3/Slc7all expression, hence inhibiting the inflammatory microenvironment, improving neurofunction recovery in cerebral I/R injury. We obtained a novel exosome, M2pep-ADSC-Exo, through engineered modification, which exhibits improved targeting capabilities toward M2 microglia. This provides a new avenue for the treatment of stroke.

**Graphical Abstract:**

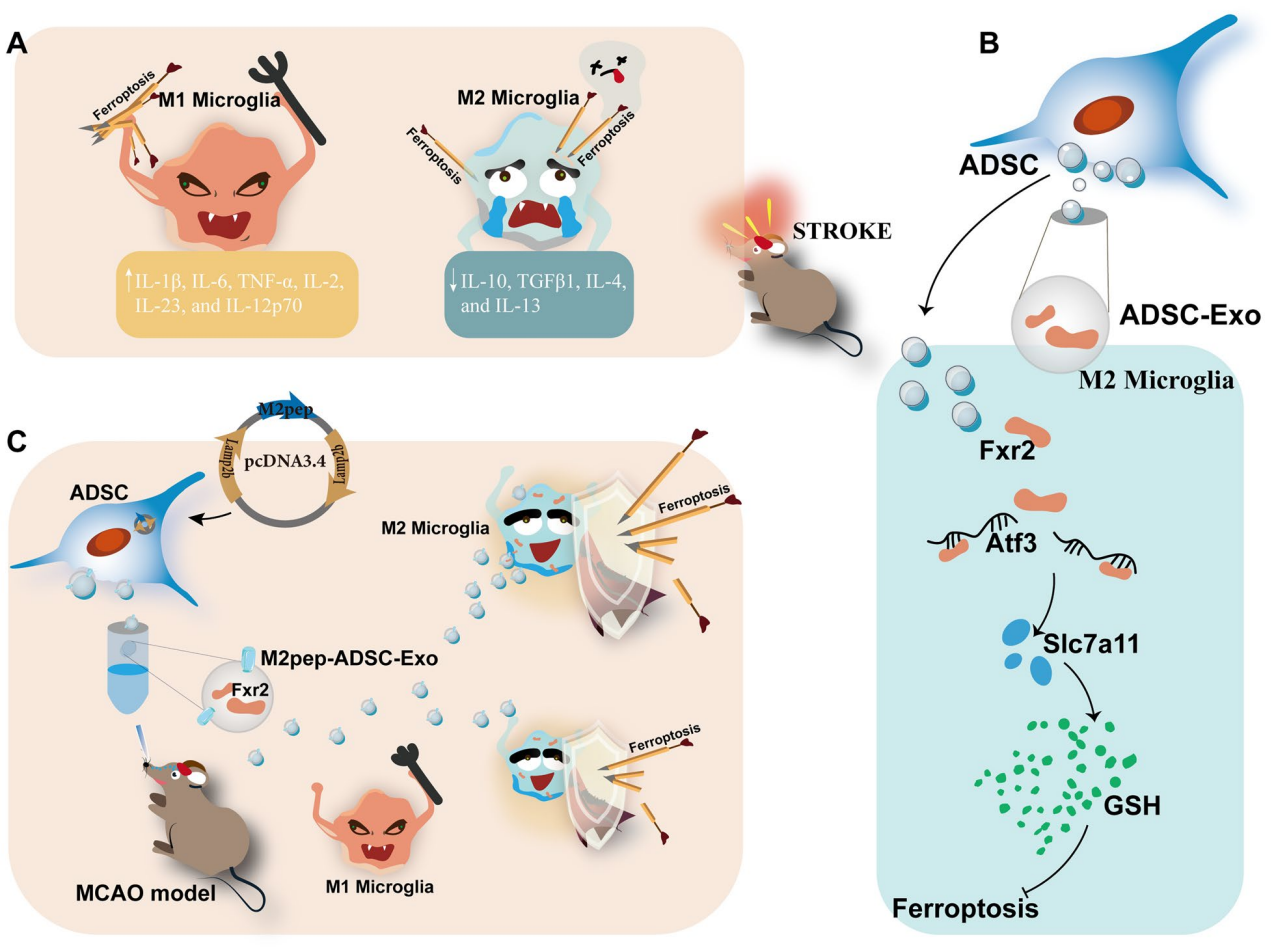

**Supplementary Information:**

The online version contains supplementary material available at 10.1186/s12951-024-02560-y.

## Introduction

Stroke, a devastating disease that affects populations worldwide, continues to be the leading cause of long-term disability and the second leading cause of death [1, 2]. The most effective treatments for ischemic stroke (IS) are intravenous thrombolysis within 3–4.5 h and arterial thrombectomy within 6 h [3, 4]. Reopening occluded blood vessels to rescue brain cells is critical to reducing disability rates following IS. However, complications from reperfusion injury undermine the clinical benefits of cerebral blood flow recovery after vascular recanalization [5]. Cerebral ischemia/reperfusion injury (I/R) refers to the phenomenon where the restoration of blood flow not only fails to recover the function of brain tissue but exacerbates the damage, further inducing various kinds of cell death in the brain [6, 7]. I/R has become a bottleneck that needs to be addressed in vascular recanalization technology.

Due to the rich phospholipid content in brain tissue, lipid peroxidation becomes a prominent feature of I/R, providing a material basis for ferroptosis in brain cells. Ferroptosis, a newly discovered form of programmed cell death, is characterized by the massive accumulation of reactive oxygen species (ROS) and iron-dependent lipid peroxidation reaching lethal levels [8, 9]. Ferroptosis plays an irreplaceable role in IS. Studies have shown that ferroptosis-related genes can be diagnostic biomarkers for IS [10]. During IS, excessive accumulation of lipid ROS leads to glutathione (GSH) depletion and GPX4 inactivation [11], while iron ion accumulation and iron-dependent lipid peroxidation also increase [12]. Structural and functional damage to the blood–brain barrier contributes to the entry of iron from the blood into the brain parenchyma [13], all of which promote ferroptosis after IS.

Additionally, a study demonstrated that the ferroptosis-related protein ACSL4 significantly influences the progression of IS [14]. Another study showed that IS mice exhibited elevated lipid peroxidation levels and reduced GSH levels. Notably, administering ferroptosis inhibitors, such as Liproxstatin-1 and Ferrostatin-1, facilitated the recovery of neurological function following I/R injury in mice [15]. Thus, ferroptosis provides a novel research direction for investigating brain cell death during I/R. Elucidating the signaling pathways of ferroptosis following stroke and interpreting the mechanisms of brain cell injury from the perspective of ferroptosis will help develop more appropriate therapeutic strategies for IS patients.

Microglia are the key focus in I/R neuroprotection research [16, 17]. Within minutes of cerebral ischemia, microglia activate, releasing inflammatory factors and modulating their phenotype and function [18]. Microglia exhibit dual roles in neuroprotection or neurotoxicity, presenting different functional states at various stages after stroke. Pro-inflammatory M1 microglia persistently increase after stroke, while anti-inflammatory M2 microglia briefly increase before rapidly declining [19]. Enhancing the proportion of anti-inflammatory M2 microglia and inhibiting the inflammatory microenvironment are critical research areas for improving I/R. Recent studies have primarily focused on inhibiting M1 microglial polarization or promoting M1-to-M2 conversion, but the mechanisms involved in altering microglial polarization are complex and challenging to implement [20]. Research has shown that different microglial subtypes exhibit varying sensitivities to the ferroptosis inducer RSL3, with M2 microglia being highly sensitive to ferroptosis and M1 microglia being resistant [21]. Given the rapid decline of M2 microglia in the acute phase of stroke, this phenomenon might be related to the high sensitivity of M2 microglia to ferroptosis. Suppose ferroptosis of existing M2 microglia can be inhibited during the acute phase, slowing down the decline in the proportion of M2 microglia and thus suppressing the inflammatory microenvironment to promote neuronal survival. In that case, it may provide innovative intervention methods for neuroprotection after IS.

The ability of drugs to be precisely delivered to the ischemic region is a critical technical bottleneck in clinical translation. Currently, none of the ferroptosis inhibitors investigated in preclinical research possess the capacity to target IS infarct foci. Thus, we must explore alternative strategies that effectively intervene in ferroptosis after IS. Exosomes (Exo) are small vesicles secreted by living cells, containing many bioactive substances [22]. They can diffuse to neighboring cells or be transported to more distant locations, altering the behavior and fate of recipient cells through mediating local and systemic intercellular communication [23, 24]. Stem cell-derived Exo, which circumvents the risks of promoting tumorigenesis and immunogenicity associated with stem cells while retaining their therapeutic benefits, has been widely studied for IS treatment [25]. The regulatory effects of stem cell-derived Exo on ferroptosis have been demonstrated in various disease models, including acute liver injury [26], acute myocardial infarction [27], murine chronic asthma [28], hepatocellular carcinoma [29], and stroke [30, 31]. Since the function of stem cell-derived Exo depends on the donor stem cells, identifying suitable donor stem cells is crucial. Considering clinical translation, donor stem cells should exhibit potent efficacy, be readily available, easily harvested, and have high acquisition rates. Adipose-derived stem cells (ADSC) are a type of stem cell capable of self-renewal and possessing multilineage differentiation potential [32]. One million ADSC can be isolated from 200 mL of human adipose tissue obtained through liposuction, with an acquisition rate of 1–2%. In contrast, the acquisition rate of bone marrow stem cells is only 0.001–0.002% [33]. Research has shown that ADSC exhibits similar therapeutic effects for IS compared to other mesenchymal stem cells [34, 35], with stronger immunoregulatory [36] and proliferative capacities [37], and slower senescence [38]. Therefore, selecting ADSC-derived Exo (ADSC-Exo) to intervene in ferroptosis after IS holds significant clinical translation potential. However, animal studies have confirmed that although Exo possesses targeting capabilities, their efficacy is not high enough [39]. Consequently, it becomes imperative to modify Exo using engineering techniques to enhance their targeting abilities further.

In this study, we observed the differential sensitivity of different subtypes of microglia to I/R-induced ferroptosis. ADSC-Exo exhibited neuroprotective effects by modulating ferroptosis in M2 microglia and suppressing the inflammatory microenvironment. We further elucidated that the regulatory impact of ADSC-Exo on ferroptosis in M2 microglia was mediated through the Fxr2/Atf3/Slc7a11 axis. To enhance the targeting capability of ADSC-Exo towards M2 microglia, we engineered M2pep-ADSC-Exo. In vitro and in vivo experiments demonstrated that M2pep-ADSC-Exo specifically targeted M2 microglia and inhibited their ferroptosis, thereby increasing the proportion of M2 microglia in the acute phase of IS.

## Materials and methods

### Animals

Healthy adult male C57/BL6 mice (10–12 weeks old) were purchased from Beijing Vital River Laboratory Animal Technology Co., Ltd. (Beijing, China). The animal facility maintained an indoor temperature of 21–26 °C with a relative humidity of 40–70%. A 12:12 h light–dark cycle was established, and the mice were provided with standard rodent food and water ad libitum. All animal experimental protocols were approved by the ethical review board of Central Hospital of Minhang District, Shanghai, China (Approval No: 2021JS-Minhang Hospital-022), and adhered strictly to the principles of animal welfare, with maximum efforts made to minimize animal suffering.

### Middle cerebral artery occlusion model (MCAO) in mice

Mice weighing 25–30 g received MCAO surgery. After intraperitoneal injection of 1% sodium pentobarbital for anesthesia, the sternocleidomastoid muscle was dissected to expose the left common carotid artery (CCA), internal carotid artery (ICA), and external carotid artery (ECA) in mice. The CCA, ICA, and ECA were ligated, and a small incision was made at the distal end of the ECA. A 6-0 nylon monofilament suture coated with silicon (Jialing Co., Ltd., Guangzhou, China) was inserted through the incision, advanced into the middle cerebral artery (MCA) via the ICA, and then secured. The ligature remained in place to induce MCA occlusion for 60 min, after which the thread was withdrawn. The residual end of the ECA was ligated, and blood flow in the CCA was restored. Regional cerebral blood flow (rCBF) was assessed in all mice with stroke through laser Doppler flowmetry (PeriFlux System 5001, Perimed, Jarfalla, Sweden). Mice that did not exhibit a minimum 75% decrease in pre-ischemia rCBF levels during the period of MCAO were not included in subsequent experiments. Imaging of rCBF was conducted utilizing laser speckle (PeriCam PSI System, Perimed, Jarfalla, Sweden). In the sham group, mice underwent the same surgical procedure but without occlusion of the middle cerebral artery by the thread.

### Cell culture

ADSCs were obtained from 8-week-old male C57/BL6 mice, following the protocol described in our previous study [30]. The surface markers of ADSCs were authenticated using the BD FACSVerse flow cytometer (BD Biosciences, USA). In brief, cells were enzymatically dissociated, suspended in PBS buffer, and subsequently incubated with the following antibodies at 4 °C for 30 min in the dark: FITC anti-mouse CD45 (Cat# 103107, Biolegend), APC anti-mouse CD34 (Cat# 128611, Biolegend), PE anti-mouse CD105 (Cat# 120407, Biolegend), and PE/Cyanine anti-mouse/human CD44 (Cat# 103029, Biolegend). The ADSCs’ capacity for multiple differentiation was evaluated using adipose mesenchymal stem cell osteogenic differentiation medium (PD-025, Pricella, Wuhan, China) and adipogenic differentiation medium (PD-027, Pricella, Wuhan, China). and the cells were subjected to staining with either Oil Red O staining solution (E607319, BBI, Shanghai, China) or alizarin red staining solution (A600144, BBI, Shanghai, China) for analysis. ADSCs were maintained in a complete medium at a temperature of 37 °C in a 5% CO2 atmosphere. The complete medium comprised L-DMEM supplemented with 10% FBS and 1% penicillin/streptomycin (Genom, Zhejiang, China), and the medium was refreshed every 2 days. BV2 microglia, N2a mouse neuroblastoma cell line, and L929 fibroblast cells were obtained from Procell Life Science & Technology Co., Ltd. (Prolong LifeTech, City, Country). They were cultured in high-glucose DMEM medium (Sigma-Aldrich, Saint Louis, MO) supplemented with 10% FBS and 1% penicillin/streptomycin at 37 °C with 5% CO_2_. The cells were passaged at a ratio of 1:3 every 2 days.

### Isolation and culture of primary microglia

Newborn C57/BL6 mice, aged 24 h, were quickly sacrificed, and their brains were collected. The brains were placed in HBSS and carefully dissected to remove the meninges. The brain tissue was then triturated, and the supernatant was collected. After centrifugation, the pellet was resuspended in an F12 medium supplemented with 10% FBS and 1% penicillin/streptomycin. The cells were seeded into T75 culture flasks with a complete culture medium. The medium was changed every 2.5 days, and after approximately 9 days, cell stratification was observed. The upper layer comprised microglia and a few oligodendrocytes, while the lower layer formed astrocytes and neurons. The microglia were collected by gentle shaking. The culture was continued for one more week before a second round of shaking was performed to collect the remaining microglia.

### Isolation, identification, and labeling of Exo

Passage five ADSCs and L929 fibroblast cells were cultured in a complete medium supplemented with exosome-depleted fetal bovine serum (FBS). Exo were obtained from the supernatant of cells using ultracentrifugation based on a previously published protocol [30]. In brief, the supernatant was collected and subjected to sequential centrifugation steps at 300*g*, 2000*g*, 10,000*g*, and 110,000*g*. The resulting pellet was resuspended in a phosphate-buffered saline (PBS) solution. The morphological features of the Exo were examined using transmission electron microscopy (TEM, Hitachi, Japan). The size distribution of the Exo was analyzed using a particle size analyzer (NanoFCM, Fujian, China). Western blot analysis assessed the protein markers of the Exo, including anti-CD63 antibody (Abcam, Cambridge, UK) and tumor susceptibility gene 101 (TSG101, Abcam, Cambridge, UK). The PKH26 red fluorescent cell linker kit (Sigma-Aldrich, Saint Louis, MO) was utilized according to the manufacturer’s instructions to label the Exo.

### Intranasal administration of Exo

Mice were placed in a supine position and subjected to isoflurane gas anesthesia while maintaining spontaneous respiration. For the Exo group, mice received intranasal administration of 2 μL PBS containing 2 μg Exo using a micropipette every 2 min. The administration was performed by instilling the solution into one nostril at a time, alternating between the two nostrils, lasting 10 min to deliver a cumulative dose of 10 μg Exo (unless otherwise specified). Mice in the Exo group received daily interventions of 10 μg Exo for 1 to 3 days after MCAO. The PBS group mice received the same volume of PBS using the same method for 3 days.

### Western blot analysis

The concentrations of protein samples were evaluated using a BCA protein assay kit (Epizyme, Shanghai, China). The primary antibodies employed in this study were as follows: anti-CD63 (ab217345, 1:1000, Abcam), TSG101 (ab125011, 1:1000, Abcam), Fxr2 (12552-1-AP, 1:1000, Proteintech), Atf3 (sc-518032, 1:500, Santa Cruz), Slc7a11 (26864-1-AP, 1:1000, Proteintech), LAMP2B (Ab13524, 1:1000, Abcam), and GAPDH (60004–1-Ig, 1:50000, Proteintech).

### RT-qPCR

Trizol reagent (Invitrogen, United States) was employed for total RNA extraction. The extracted RNA samples were then reverse-transcribed into cDNA using the PrimeScriptTM RT reagent Kit (TAKARA, Japan). RT-qPCR was carried out on a Light Cycler thermal cycler system (Bio-Rad, United States) using SYBR® Premix Ex Taq™ II (TAKARA, Japan). For mRNA normalization, GPADH was used as the internal control. The relative expression was compared to the control group. The sequences of the primers used in the present study are shown in Table 1.

**Table 1:** Primers for mRNA real-time polymerase chain reaction

Gene	Primer (5′ to 3′)
Atf3	F: AGCGAAGACTGGAGCAAAATG R: CATCCGATGGCAGAGGTGTT
Fxr2	F: AGCTCTTTATTCTGTCAACCACA R: GCCAGCTGCTTACTTGTCTCT
CD32	F: AATCCTGCCGTTCCTACTGATC R: GTGTCACCGTGTCTTCCTTGAG
CD206	F: CAAGGAAGGTTGGCATTTGT R: CCTTTCAGTCCTTTGCAAGC
iNOS	F: CAAGCACCTTGGAAGAGGAG R: AAGGCCAAACACAGCATACC
Arg1	F: TCACCTGAGCTTTGATGTCG R: CTGAAAGGAGCCCTGTCTTG
Cstf2t	F: ATCATGTCGAGTTTGGCGGT R: AGAAGCCATAACCCTTGGGC
Upf1	F: GACGTGGCACTCTCACAAGA R: GCTCTTCCTCCGCTACCTTC
Numa1	F: GCCGACTGAGAAGAAACCCA R: CACTCCAACTCACCGGACTC
Rbm10	F: CAGCTCTCCACCATCGAAGC R: ATGCGACTGCCTTCATTGGA
GAPDH	F: ATCATCAGCAATGCCTCCTG R: ATGGACTGTGGTCATGAGTC

### Immunofluorescence staining

After administering anesthesia to the mice, their brains were extracted following perfusion with 4% paraformaldehyde. The brains were dehydrated using sucrose and embedded in OCT compound (Sakura, Japan) before rapidly frozen at -20℃. Subsequently, the brains were coronally sliced into sections with a thickness of 30 μm. The brain sections underwent a triple wash with PBST and were incubated overnight at 4℃ with specific antibodies IBA1 (016-26721, 1:200, Wako) and CD206 (AF2535, 1:500, R&D Systems). Following another round of triple washes with PBST, the brain sections were exposed to different secondary antibodies (Invitrogen, United States) for 1 h at room temperature. After a final triple wash with PBST, the brain sections were mounted using a DAPI-containing mounting medium (southenbiotech, United States). Immunofluorescence microscopy (Keyence, Shanghai, China) was used to observe and capture images of three sections of each mouse.

### Oxygen–glucose deprivation (OGD)

To simulate ischemia and hypoxia conditions in vitro, OGD treatment was implemented. This involved replacing the complete medium with glucose-free DMEM and subjecting cells to a hypoxic chamber of 95% N2 and 5% CO2. Subsequently, the cells were cultured in the complete medium under normoxic conditions. The control group of cells did not undergo OGD treatment.

### CCK8

The cells were plated in 96-well plates at a density of 5 × 10^3^ cells per well and incubated at 37 °C with 5% CO_2_ for 24 h. Following the respective treatments, a fresh complete medium supplemented with 10% CCK8 solution (Beyotime, Shanghai, China) was added to each well, and the cells were further incubated for 2 h. Subsequently, each well's absorbance value (OD value) was determined using a microplate reader at a wavelength of 450 nm.

### Lipid peroxidation malondialdehyde (MDA) assay

MDA assay kits (Beyotime, Shanghai, China) were utilized to evaluate the level of lipid peroxidation. The brain tissue of mice or cells was lysed with RIPA lysis buffer (Beyotime, Shanghai, China). Following centrifugation at 12000*g* for 10 min, the supernatant was collected. The supernatant was combined with a working solution in a 2:1 ratio and heated at 100 °C for 15 min. After centrifugation at 1000*g* for 10 min, 200 μL of the supernatant mixture was added to 96-well plates, and the OD value was measured at a wavelength of 532 nm using a microplate reader. The concentration of MDA was determined by considering the MDA content and protein level in each sample.

### Flow cytometry with the C11-BODIPY probe

Cellular lipid peroxidation was measured using the live cell analysis reagent C11-BODIPY 581/591 (Thermo Fisher Scientific, MA, USA). Cells were seeded at a density of 2 × 10^5^ cells per well in 6-well plates and cultured at 37 °C with 5% CO_2_ for 24 h. Following the respective treatments, the cells were washed twice with PBS buffer and incubated with a staining solution of C11-BODIPY (10 μM) in the dark for 30 min at 37 °C. Subsequently, the cells were collected using a 0.25% trypsin solution, washed twice with PBS buffer, and resuspended in PBS buffer. The cells were promptly analyzed using a flow cytometer.

### FerroOrange probe

The intracellular ferrous iron level (Fe_2_ +) in microglia was assessed using the FerroOrange Probe (F374, Dojindo). Microglia were plated at a density of 1 × 10^4^ cells/well in 96-well plates and incubated at 37 °C with 5% CO_2_ for 24 h. Following various treatments, the cells were washed twice with PBS buffer and then exposed to FerroOrange staining solution (1 μM) in the dark for 30 min at 37 °C. Subsequently, the cells were analyzed using a colorimetric microplate reader (Ex: 543 nm, Em: 580 nm).

### Inflammatory cytokine array

Three days after the surgery, mice were sacrificed, and cortical brain tissues from ischemic penumbra were extracted. The levels of 18 cytokines were measured using the QAM-TH17 inflammatory cytokine array (Raybiotech, USA).

### Conditioned medium assay

Primary microglia were seeded at a density of 3 × 10^5^ cells per well in a 6-well plate for culturing 24 h. After performing OGD, different interventions were applied during the reoxygenation phase for 24 h. The culture medium from different groups was collected and centrifuged at 400*g* and 4 °C for 5 min to obtain the supernatant, called the conditioned medium. N2a cells were seeded at a density of 5 × 10^3^ cells per well in a 96-well plate. The cells were cultured in a high-glucose DMEM medium containing 10% FBS and 1% penicillin/streptomycin under 37 °C and 5% CO2 conditions. After 24 h, the original culture medium was removed, and 100 μL of fresh or conditioned medium was added. After 24 h of incubation, cell viability was assessed using a CCK-8 assay.

### Elisa assay

Primary microglia were seeded at a density of 3 × 10^5^ cells per well in a 6-well plate and cultured for 24 h. After inducing OGD, various interventions were applied during the reoxygenation phase for 24 h. The culture medium from each group was collected and centrifuged at 400*g* and 4 °C for 5 min to obtain the supernatant. ELISA kits for TNF-α (RK00027, Abclonal) and IL-10 (RK00016, Abclonal) were used to perform ELISA.

### Polarization of microglia into M1 and M2 Phenotypes

Microglia were polarized into M1-like microglia by stimulation with lipopolysaccharide (LPS, E.Coli 0111:B4, 100 ng/mL) combined with interferon-gamma (IFN-γ, 20 ng/mL) for 24 h. Similarly, M2-like microglia polarization was achieved by treating the cells with interleukin-4 (IL-4, 20 ng/mL) combined with transforming growth factor-beta (TGF-β, 20 ng/mL) for 24 h.

### Isolation of microglia from adult mouse brain

Three days after the surgery, mice were anesthetized with a 1% sodium pentobarbital intraperitoneal injection. After saline cardiac perfusion, cortical brain tissues were extracted. The brain tissues were dissociated using an adult mouse brain dissociation kit (130-107-677, Miltenyi). Subsequently, microglia were isolated using CD11b microglia MicroBeads (130-093-634, Miltenyi).

### Next-generation sequencing

Second-generation sequencing was performed on ADSC-Exo, L929-Exo, and isolated adult mouse microglia. Sequencing was performed using the Illumina NovaSeq™ 6000 platform with paired-end (PE) 150 bp sequencing mode.

### Actinomycin D experiment

Primary microglia were seeded at a density of 3 × 10^5^ cells per well in a 6-well plate for 24 h, and then microglia polarization into M2 microglia was induced. Subsequently, the cells were treated with either PBS or ADSC-Exo. Simultaneously, a transcription inhibitor, actinomycin D, was added at a concentration of 5 μM. At different time points (0 h, 3 h, and 6 h) following the addition of actinomycin D, the cells from each group were collected for RNA extraction. The mRNA levels of target genes were assessed using RT-qPCR.

### RNA binding protein immunoprecipitation (RIP) assay

The RIP experiment used the PureBinding® RNA Immunoprecipitation Kit (P0101, Gisai Biotech, China). Primary microglia were cultured in 10 cm culture dishes at a seeding density of 5 × 10^6^ cells per dish. After 24 h, the cells were induced to acquire the M2 phenotype. Subsequently, M2 microglia were collected through pancreatic enzyme digestion following the instructions provided in the kit. The protein and mRNA levels of the target were assessed using Western blotting and RT-qPCR, respectively, to determine their expression levels.

### Construction of stable cell line overexpressing Fxr2

BV2 cells were infected with Fxr2 lentivirus containing GFP fluorescence marker and Puromycin resistance in a 24-well plate. A preliminary experiment was conducted to determine the infection multiplicity (MOI = 500) and the lethal concentration of Puromycin (4 μg/mL). On day 1, BV2 cells were seeded at 5 × 10^4^ cells per well in the 24-well plate and incubated overnight at 37 °C. On day 2, the virus was diluted in a fresh culture medium containing 5 μg/mL Polybrene before infection. The virus solution was added to the cells and incubated overnight at 37 °C. After 16–24 h of infection, the cells were switched to a fresh culture medium. After 72 h of incubation, the cells were cultured in a medium containing 4 μg/mL Puromycin, with regular medium changes every 3 days. The control group, which was not infected with the virus, was eliminated, while the virus-infected group showed no cell death. The antibiotic concentration was reduced to 2 μg/mL for further selection and expansion of the cells. Concurrently, cells were collected for RT-qPCR identification and cells with confirmed expected identification results were cryopreserved.

### siRNA transfection/genes overexpression

The siAtf3 and siFxr2 were acquired from Genomeditech Co., Ltd. (Shanghai, China) and transfected using Lipofectamine 3000 reagent (Invitrogen, Carlsbad, CA, USA). To achieve overexpression of Atf3 or Fxr2, the genome from Genomeditech (Shanghai, China) was integrated into the BV2 genome using lentiviral transfection with a multiplicity of infection (MOI = 500).

### GSH/GSSH assay

The Glutathione Assay Kit (S0053, Beyutime) was employed in this study. Tissue or cellular samples were collected and prepared following the manufacturer’s instructions. Subsequently, the samples were added to a 96-well plate. Then, 150 μL of the total glutathione detection working solution was added, and the mixture was incubated at room temperature for 5 min. Finally, 50 μL of 0.5 mg/mL NADPH solution was added, thoroughly mixed, and incubated for 25 min. The absorbance was measured using a colorimetric microplate reader at a wavelength of 412 nm. The calculation formula is as follows: GSH = Total Glutathione−GSSG × 2.

### Plasmid transfection

P3 passage of ADSC (8 × 10^5^ cells/well) were seeded in 10 cm dishes with 10 mL antibiotic-free medium. After 24 h of incubation at 37 °C and 5% CO2, 10 μg of plasmid DNA was diluted in 1250 μL Opti-MEM and gently mixed. Simultaneously, 25 μL of lipofectamineTM3000 was diluted in 1250 μL Opti-MEM and incubated for 5 min. The diluted transfection reagent and plasmid DNA were mixed and set for 20 min. Then, the transfection complex (2500 μL per well) was added to the cells, followed by gentle shaking. After 72 h of transfection, the cells were collected for further experiments.

### Flow cytometry analysis of M1 and M2 microglia

Resuspend cells in 1 mL of staining buffer to adjust the cell concentration to 2 × 10^7^/mL. Transfer 100 μL of the suspension to a flow cytometry tube and set up a single-stained control tube, fluorescence minus one (FMO) control tube, blank tube, and sample tubes. Add 0.1 μL of FVS (in FVS single-stained control and sample tubes), vortex gently to mix, and incubate at 4 °C in the dark for 30 min. Centrifuge at 350*g* for 5 min at 4 °C, and discard the supernatant. Add 100 μL of staining buffer containing 2 μL of Fc blocker (for all tubes), vortex gently to mix, and incubate at 4 °C in the dark for 15 min. Centrifuge at 350*g* for 5 min at 4 °C, and discard the supernatant. Add 100 μL of staining buffer containing PE anti-mouse CD45 (157603, Biolegend), BV421 anti-mouse/human CD11B (101251, Biolegend), and APC anti-mouse CD86 (558703, BD) antibodies, vortex gently to mix, and incubate at 4 °C in the dark for 30 min. Wash once with 1 mL of staining buffer, centrifuge at 350*g* for 5 min at 4 °C, and discard the supernatant. For cell membrane permeabilization, resuspend cells in 100 μL of staining buffer, add 250 μL of Fixation/Permeabilization solution, and incubate at 4 °C for 20 min. Terminate the permeabilization by adding 1 mL of 1 × Perm/wash buffer, centrifuge at 350*g* for 5 min at 4 °C, and discard the supernatant. Add 100 μL of staining buffer containing PE/Cyanine7 anti-mouse CD206 (141720, Biolegend) antibody, gently mix, and incubate at 4 °C in the dark for 30 min. Wash once with 2 mL of PBS, and centrifuge at 350*g* for 5 min at 4 °C. Resuspend cells in 500 μL of PBS and analyze using flow cytometry.

### Triphenyltetrazolium chloride (TTC) staining

On the third day following reperfusion, the mice were swiftly euthanized, and their brains were promptly extracted and sliced coronally into 2 mm thick sections. These brain slices were submerged in a 2% TTC saline solution and then incubated for 15 min at 37 °C. Subsequently, the brain slices were fixed in a 10% formalin solution. The infarcted regions appeared white, while normal brain tissue exhibited a red coloration. High-resolution photographs of these sections were captured. The extent of infarction in each section was quantified by assessing the change in coloration. Infarct size was determined through digital planimetry of the slices using Image J software.

### Hematoxylin and eosin (HE) staining

The sections underwent staining with HE. The images were visualized using a 20 × laser scanning confocal microscope (Olympus FV1200, Tokyo, Japan).

### Neurobehavioral tests

To assess the neurobehavioral function of the mice, the modified neurological severity score (mNSS), the open-field test, and the foot fault test were employed. At 1, 3, 7, and 14 days following MCAO, the mNSS was utilized to evaluate the neurological deficits of the mice. The total scores on this scale ranged from 0 to a maximum of 14, with severity categorized as follows: mild (1–5 scores), moderate (6–10 scores), and severe (11–15 scores). Furthermore, the motor function of the mice at 3 days after MCAO was evaluated using the open-field test. Each mouse was placed in the center of a plastic box measuring 50 × 50 cm and allowed to explore the area for 5 min. The movement trajectories of the mice were recorded and analyzed using a video tracking system (ANYmaze, Stoelting, IL, USA) to determine the total distance covered by the mice within the open field. The foot fault test was employed to evaluate the grasping function of the mice's affected limb at 7 and 14 days post-MCAO. During this assessment, the mice consistently traversed a 1-m-long horizontal ladder, performing three repetitions. The entire procedure was captured on camera, and the percentage of partially placed steps to the total number of steps taken by the affected limb was subsequently calculated.

### Statistical analysis

All data in this experiment are presented as mean ± standard deviation (mean ± SD) and analyzed using GraphPad Prism 9.0 software (La Jolla, CA, USA). For comparisons between the two groups, a t-test was employed. One one-way ANOVA analysis followed by Tukey’s multiple comparison test was performed for comparisons among various groups. All tests were two-tailed, and p < 0.05 was considered statistically significant.

## Results

### The ferroptosis inhibitor can modulate inflammatory response in MCAO mice

We successfully isolated primary ADSCs (Supplementary Fig. 1A–D) and their secreted exosomes (ADSC-Exo). As shown in Supplementary Fig. 2A, the laser Doppler flowmetry indicated that the rCBF decreased over 75% during the period of MCAO. The laser speckle imaging revealed that the rCBF was blocked during ischemia and slightly recovered after reperfusion (Supplementary Fig. 2B). These results indicated that the MCAO mice model was successfully established. Then, different doses of PKH-26-labeled ADSC-Exo were administered to MCAO mice via intranasal delivery immediately after modeling. The mice were sacrificed 6 h after administration, and brain tissues were obtained for immunofluorescence analysis. As shown in Supplementary Fig. 3A–F, the signal of PKH-26-labeled ADSC-Exo in the ischemic penumbra of mice increased with higher doses of ADSC-Exo, reaching the maximum cellular uptake at a dose of 10 μg. These results indicate that 10 μg is the optimal dosage for intranasal administration of ADSC-Exo.

**Fig. 1:**
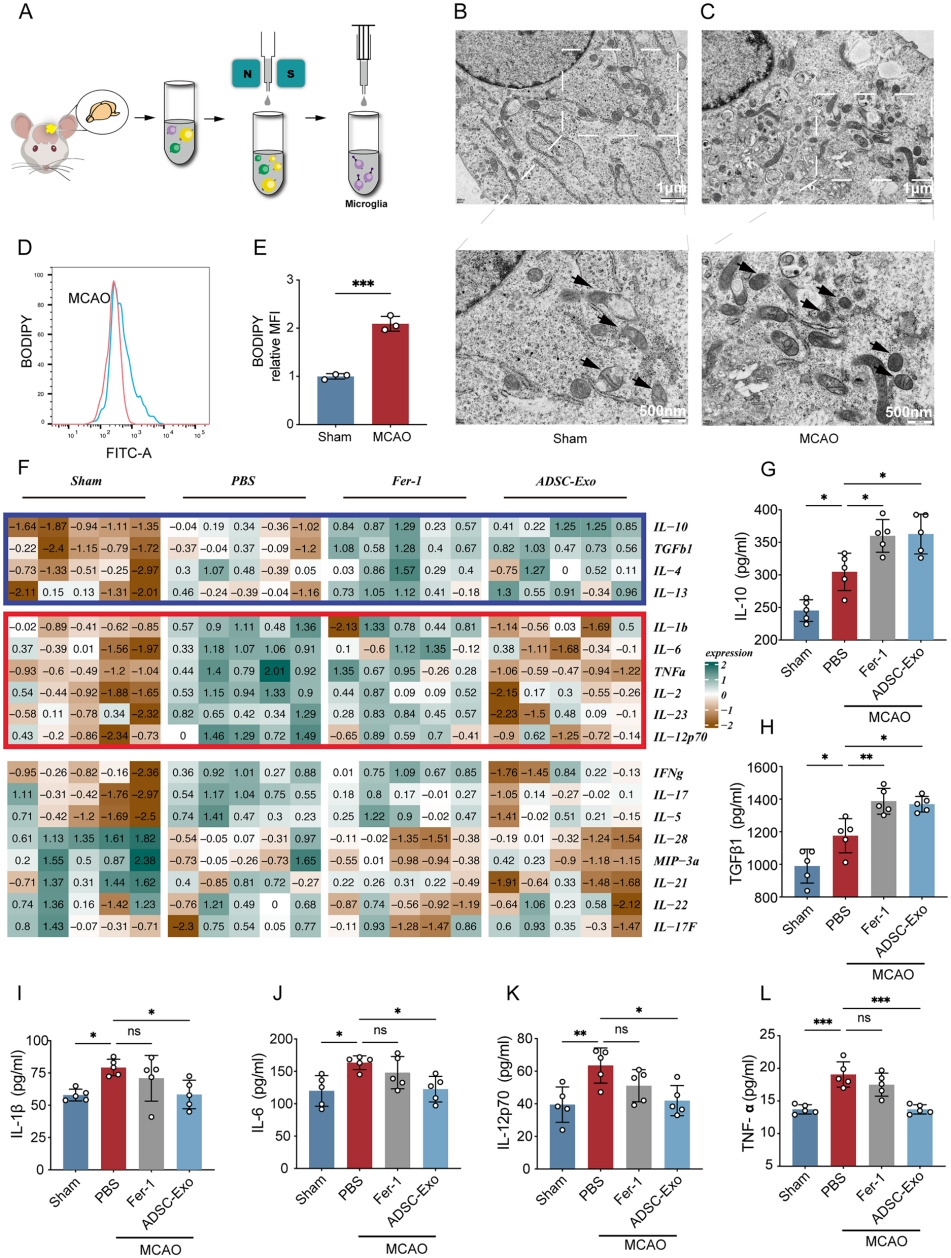
The ferroptosis inhibitor can modulate inflammatory response in MCAO mice. **A** Isolation of microglia from the cortical brain tissue of mice at 3 days post-MCAO. **B** Transmission electron microscopy (TEM) observation of mitochondrial morphology in microglia from sham-operated mice (Sham group), scale bar = 1 μm; white dashed box shows the magnified image with black arrows indicating mitochondria, scale bar = 500 nm. **C** TEM observation of mitochondrial morphology in microglia from MCAO model mice (MCAO group), scale bar = 1 μm; white dashed box shows the magnified image with black arrows indicating mitochondria, scale bar = 500 nm. **D**, **E** C11-BODIPY 581/591 probe assessment of lipid ROS levels in microglia from mouse cortical brain tissue, n = 3. **F** Intranasal administration of PBS, Fer-1 (10 ng/kg), or ADSC-Exo (1000 μg/mL, 10 μg/day, 3 days) was performed, and the levels of inflammatory cytokines in the cortical brain tissue of mice were assessed using an inflammatory cytokine array 3 days after MCAO. The red box represents inflammation-related factors associated with M1 microglia, while the blue box represents inflammation-related factors associated with M2 microglia, n = 5. **G** Quantitative analysis of the inflammatory cytokine IL-10, **H** TGFβ1, **I** IL-1β, **J** IL-6, **K** IL-12p70, **L** TNF-α levels, n = 5. *p < 0.05; **p < 0.01; ***p < 0.001

Using CD11b MicroBeads-based separation, microglia were isolated from the ischemic penumbra cortical brain tissue of mice at 3 days post-MCAO, and ferroptosis in microglia was assessed through transmission electron microscopy and C11-BODIPY 581/591 fluorescent probe (Fig. 1A). Compared to the Sham group, the microglia in the MCAO group exhibited reduced mitochondrial size, increased mitochondrial membrane density, and decreased or even absent mitochondrial cristae (Fig. 1B, C). These findings are consistent with the ultrastructural characteristics associated with ferroptosis. C11-BODIPY assay revealed an elevated relative fluorescence intensity in the FITC channel for microglia in the MCAO group (p < 0.001, Fig. 1D E), indicating enhanced lipid peroxidation in these cells. These findings suggest that I/R triggers ferroptosis in microglia. Mice were then randomly assigned to a PBS intervention group, a ferroptosis inhibitor Fer-1 intervention group, and an ADSC-Exo intervention group, with a Sham control group also established. Cortical brain tissue from the ischemic side was collected 3 days after MCAO for cytokine array analysis. As shown in Fig. 1F–L, M1microglia-related cytokines (IL-1β, IL-6, TNF-α, IL-2, IL-23, and IL-12p70) and M2 microglia-related cytokines (IL-10, TGFβ1, IL-4, and IL-13) were increased after MCAO. The level of M2 microglia-related cytokines was further increased with the intervention of Fer-1 or ADSC-Exo, while Fer-1 did not regulate the level of M1 microglia-related cytokines. This experiment suggests that Fer-1 may inhibit ferroptosis of M2 microglia without significantly affecting M1 microglia.

### Different subtypes of microglia exhibit distinct sensitivities to I/R-induced ferroptosis

**Fig. 2:**
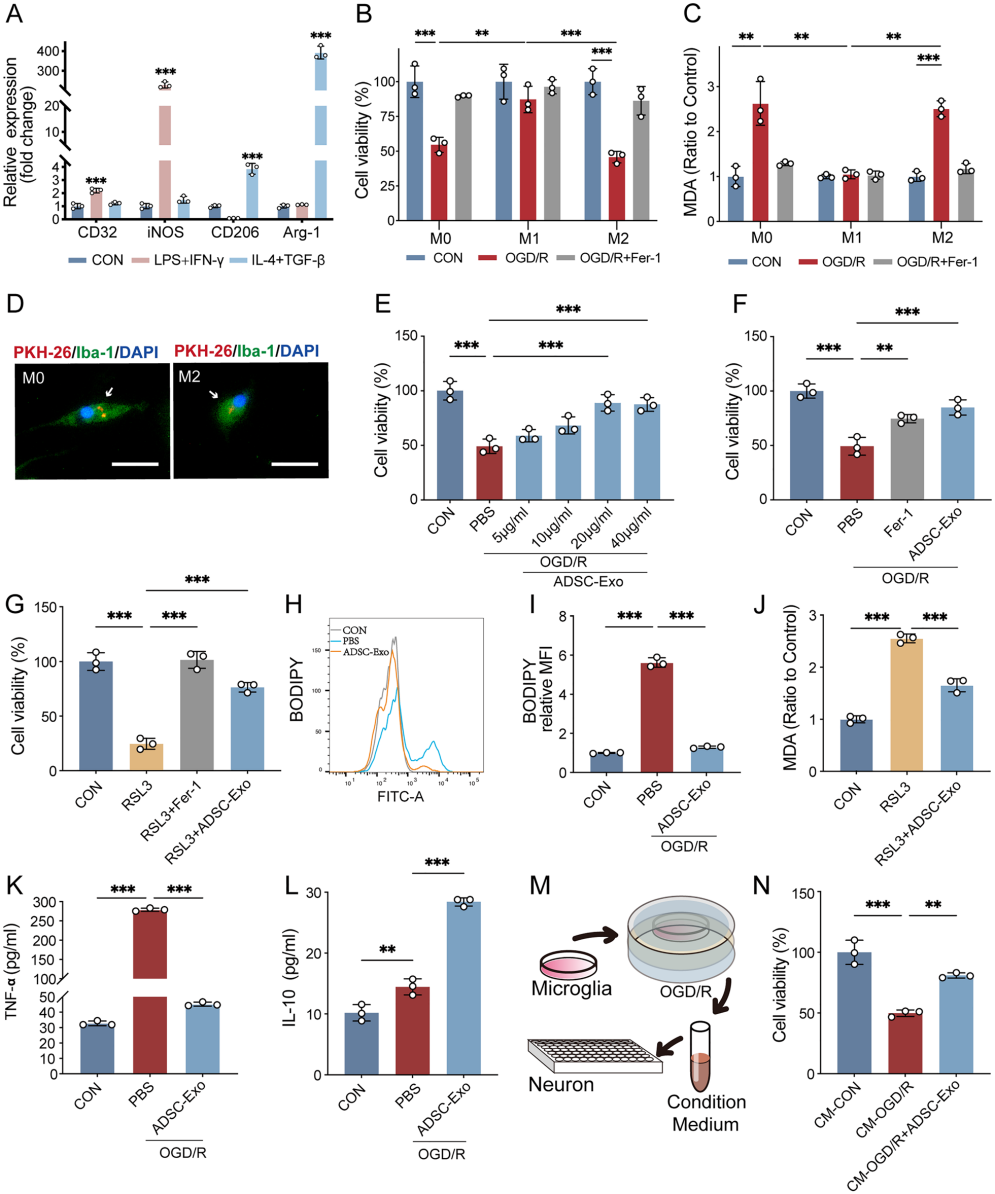
Different subtypes of microglia exhibit distinct sensitivities to I/R-induced ferroptosis, and ADSC-Exo effectively decreases the susceptibility of M2 microglia to ferroptosis, suppressing the inflammatory microenvironment and promoting neuronal survival. **A** After 24 h of induction with LPS (100 ng/mL) + IFN-γ (20 ng/mL) or IL-4 (20 ng/mL) + TGF-β (20 ng/mL), RT-qPCR was used to detect the expression levels of M1 and M2 subtype markers in primary microglia of different intervention groups, n = 3. **B** CCK-8 and **C** malondialdehyde (MDA) assays were employed to assess the sensitivity of different subtypes of microglia to ferroptosis induced by OGD/R (OGD/R 3/24 h) and the rescue effect of the ferroptosis inhibitor Fer-1 (500 nM, 6 h) on different subtypes of microglia, n = 3. **D** Immunofluorescence observation of M0 and M2 microglia (green) taking up PKH-26-labeled ADSC-Exo (red) after 6 h of co-culture in vitro; scale bar = 10 μm. **E** CCK-8 assay evaluating the effects of different concentrations of ADSC-Exo (24 h) on the survival rate of M2 microglia after OGD/R (3/24 h); n = 3. **F** CCK-8 assay evaluating the effects of ADSC-Exo (20 μg/mL, 24 h) and Fer-1 (500 nM, 24 h) on the survival rate of M2 microglia after OGD/R; n = 3. **G** CCK-8 assay evaluating the effects of ADSC-Exo and Fer-1 on the survival rate of M2 microglia after RSL-3 (500 nM, 6 h) treatment; n = 3. **H**, **I** C11-BODIPY assay evaluating the effects of ADSC-Exo on lipid ROS levels in M2 microglia after OGD/R; n = 3. **J** Malondialdehyde (MDA) assay detecting the impact of ADSC-Exo on ferroptosis in M2 microglia after RSL-3 treatment; n = 3. **K** ELISA assay evaluating the effects of ADSC-Exo on TNF-α levels in the culture medium of microglia after OGD/R; n = 3. **L** ELISA assay assesses the effects of ADSC-Exo on IL-10 levels in the culture medium of microglia after OGD/R; n = 3. **M** Schematic diagram of the conditioned medium experiment. **N** CCK-8 assay evaluating the effects of conditioned medium from microglia treated with PBS or ADSC-Exo (20 μg/mL) for 24 h on the survival rate of N2a neurons (treated for 24 h); n = 3. **p < 0.01; ***p < 0.001

To determine whether different subtypes of microglia display distinct sensitivities to ferroptosis following I/R, we first induced primary microglia to polarize into different subtypes in vitro. Then, we treated them with ferroptosis inducers or OGD/R. RT-qPCR results showed that primary microglia treated with LPS and IFN-γ displayed significantly increased levels of M1 microglial markers CD32 and iNOS compared to the control group (p < 0.001). In contrast, primary microglia treated with IL-4 and TGF-β exhibited significantly increased levels of M2 microglial markers CD206 and Arg-1 compared to the control group (p < 0.001, Fig. 2A). This indicates that we successfully polarized primary microglia into M1 and M2 subtypes using LPS and IFN-γ or IL-4 and TGF-β, respectively.

Next, we divided the different subtypes of microglia into a control group (CON), a ferroptosis inducer RSL3-treated group (RSL3), and an RSL3 plus Fer-1 group (RSL3 + Fer-1) to assess cell viability and lipid peroxidation levels. As shown in Supplementary Fig. 4A, both M0 and M2 microglia displayed significantly reduced cell viability in the RSL3 group compared to the CON group (p < 0.001), which Fer-1 rescued. In contrast, there was no significant difference in cell viability between the RSL3 and CON groups in M1 microglia (p > 0.05). Consistent results were obtained in the MDA assay (Supplementary Fig. 4B), with M0 and M2 microglia displaying significantly increased MDA levels in the RSL3 group compared to the CON group (p < 0.001). In M1 microglia, there was no significant difference in MDA levels between the RSL3 and CON groups (p > 0.05).

We further divided the different subtypes of microglia into the CON group, OGD/R group, and OGD/R + Fer-1 group to investigate the differences in sensitivity to ferroptosis induced by OGD/R among different microglial subtypes. As shown in Fig. 2B, the cell viability of M0 and M2 microglia in the OGD/R group was significantly reduced compared to the CON group (p < 0.001), and Fer-1 could rescue it. However, there was no statistically significant difference in the cell viability between the OGD/R group and the CON group in M1 microglia (p > 0.05). MDA assay revealed that MDA levels in M0 (p < 0.01) and M2 microglia (p < 0.001) in the OGD/R group were significantly higher than those in the CON group. In contrast, there was no significant difference in MDA levels between the OGD/R and the CON groups in M1 microglia (p > 0.05, Fig. 2C). Furthermore, FerroOrange probe detection indicated that the intracellular Fe2 + level in M1 microglia did not show a significant increase after OGD/R, while ADSC-Exo did not exert a significant effect on Fe2 + levels in M1 microglia (Additional file 4C). These experiments demonstrated that different microglial subtypes exhibit varying sensitivities to ferroptosis induced by I/R, with the M1 subtype being insensitive and the M2 subtype highly sensitive.

### ADSC-Exo effectively decreased the susceptibility of M2 microglia to ferroptosis, suppressing the inflammatory microenvironment and promoting neuronal survival

To explore whether ADSC-Exo can reduce the sensitivity of M2 microglia to ferroptosis, we further induced microglia polarization to the M2 subtype. We treated them with ferroptosis inducer or OGD/R intervention, observing the therapeutic effect of ADSC-Exo. As shown in Fig. 2D, PKH-26-labeled ADSC-Exo was taken up by primary M0 and M2 microglia in vitro, providing a feasible basis for ADSC-Exo intervention. CCK-8 assay showed that the cell viability of M2 microglia after OGD/R increased with the elevation of ADSC-Exo concentration, with the most significant effect observed at 20 μg/mL (p < 0.001, Fig. 2E). This concentration was determined to be optimal and used in subsequent experiments. Meanwhile, under the same conditions, the presence of ferroptosis in M2 microglia after OGD/R or RSL3 treatment and the anti-ferroptosis ability of ADSC-Exo were confirmed by CCK-8 assay (Fig. 2F, G). C11-BODIPY probe detection showed that ADSC-Exo could reduce lipid ROS levels in M2 microglia caused by OGD/R (p < 0.001, Fig. 2H, I); MDA assay revealed that ADSC-Exo could reduce MDA levels in M2 microglia induced by RSL3 (p < 0.001, Fig. 2J).

To further explore whether the regulatory effect of ADSC-Exo on ferroptosis in M2 microglia could suppress the inflammatory microenvironment and subsequently affect surrounding neurons, we conducted conditioned medium experiments. Microglia were divided into CON, PBS, and ADSC-Exo groups, and the culture medium was collected after OGD/R for inflammation factor detection. Elisa assay found that TNF-α (p < 0.001) and IL-10 (p < 0.01) increased in the PBS group after OGD/R. At the same time, TNF-α decreased (p < 0.001), and IL-10 further increased (p < 0.001) in the ADSC-Exo group compared to the PBS group, indicating that ADSC-Exo could suppress the inflammatory microenvironment of OGD/R-treated microglia (Fig. 2K, L). As shown in Fig. 2M, N, further treatment of N2a cells with conditioned medium from different intervention groups showed that the conditioned medium from the OGD/R group reduced N2a cell viability compared to the CON group (p < 0.001). In contrast, the conditioned medium from microglia treated with ADSC-Exo increased N2a cell viability compared to the PBS group (p < 0.01). These results suggest that ADSC-Exo can reduce the ferroptosis sensitivity of M2 microglia, increase anti-inflammatory factors in the inflammatory microenvironment, and improve the cell viability of surrounding neurons.

### Fxr2 in ADSC-Exo can bind to Atf3 mRNA in M2 microglia

**Fig. 3:**
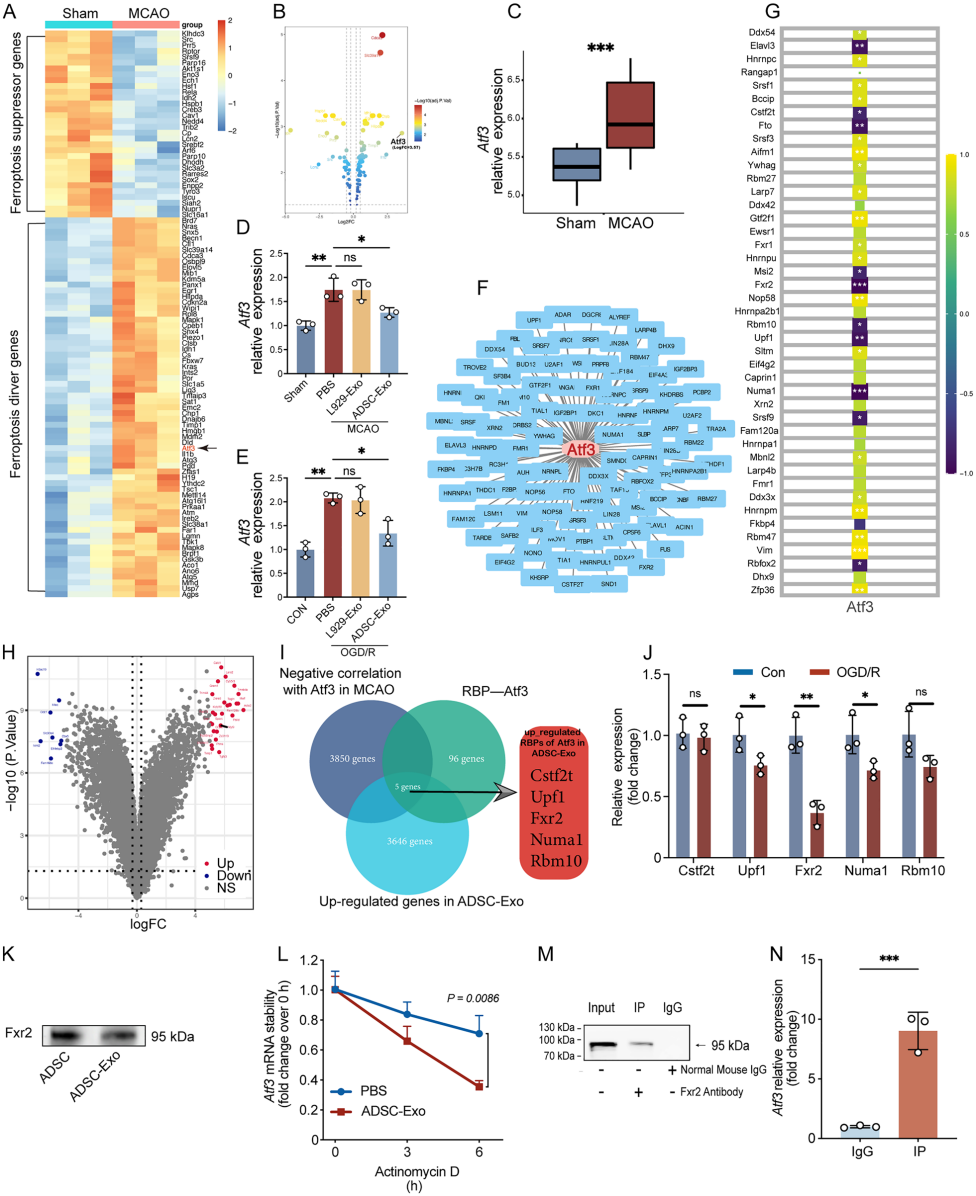
CD11b MicroBeads sorting combined with second-generation sequencing revealed Atf3 as a key gene in ferroptosis of M2 microglia, and ADSC-Exo-contained Fxr2 can bind to Atf3 mRNA in M2 microglia. **A** CD11b MicroBeads sorting was used to isolate microglia from the ischemic penumbra cortex of mice, followed by second-generation sequencing. The heatmap shows that 97 differentially expressed genes related to ferroptosis were identified in cortical microglia of Sham and MCAO mice, including 65 pro-ferroptosis genes and 32 anti-ferroptosis genes, n = 3. **B** The volcano plot shows that Atf3 is the gene with the largest difference in expression, n = 3. **C** The expression difference of Atf3 in cortical microglia of Sham and MCAO mice, n = 3. **D** RT-qPCR was used to assess the differences in Atf3 expression in ischemic penumbra cortex microglia of mice treated with PBS (10 μL/day, 3 days), L929-Exo (1000 μg/mL, 10 μL/day, 3 days), or ADSC-Exo (1000 μg/mL, 10 μL/day, 3 days), n = 3. **E** RT-qPCR was used to assess the differences in Atf3 expression in M2 microglia treated with PBS, L929-Exo (20 μg/mL, 24 h), or ADSC-Exo (20 μg/mL, 24 h), n = 3. **F** The Starbase database identified 101 RBPs that can bind to Atf3. **G** Correlation analysis showed that 43 RBPs were related to the changes in Atf3 expression levels in microglia after MCAO. **H** L929-Exo and ADSC-Exo were collected for second-generation sequencing, and the volcano plot showed that 3,651 genes were highly expressed in ADSC-Exo compared to L929-Exo, n = 3. **I** Venn diagram identified 5 candidate RBPs. **J** RT-qPCR assessed the expression changes of the 5 candidate RBPs in M2 microglia after OGD/R (3/24 h), n = 3. **K** Western blot assessed Fxr2 protein levels in ADSC and ADSC-Exo. **L** Upon adding PBS or ADSC-Exo (20 μg/mL) to the culture medium of M2 microglia along with 5 μM actinomycin D, the effect of ADSC-Exo on the stability of Atf3 mRNA in M2 microglia was evaluated, n = 3. **M**, **N** RIP experiment assessed the binding of Fxr2 protein to Atf3 mRNA, n = 3. *p < 0.05; **p < 0.01; ***p < 0.001.

To clarify the molecular mechanism of ADSC-Exo reducing the ferroptosis sensitivity of M2 microglia, we sorted microglia from the ischemic side of the brain tissue in Sham and MCAO mice using CD11b microglia MicroBeads and performed second-generation sequencing. 97 differentially expressed genes related to ferroptosis were screened, including 65 pro-ferroptosis genes and 32 anti-ferroptosis genes (Fig. 3A). As shown in the volcano plot in Fig. 3B, Atf3 is the ferroptosis-related gene with the most significant difference in expression between the Sham and MCAO groups. Sequencing data further showed that the expression level of Atf3 in microglia of the MCAO group was significantly higher than that in the Sham group (p < 0.001, Fig. 3C). Based on the results of the second-generation sequencing, we performed validation experiments in vivo and in vitro. As shown in Fig. 3D, mice were divided into Sham and MCAO groups. Based on the intervention measures, the MCAO group was further divided into PBS, exosome control (L929-Exo), and ADSC-Exo groups. Brain tissues were collected 3 days after surgery for CD11b MicroBeads sorting of microglia. RT-qPCR results showed that Atf3 expression in microglia significantly increased after MCAO (p < 0.01). Compared with the PBS group, Atf3 levels in the L929-Exo group did not change considerably as a negative control, while Atf3 levels in the ADSC-Exo group significantly decreased (p < 0.05). As shown in Fig. 3E, primary microglia were induced into M2 microglia and divided into CON and OGD/R groups, with the OGD/R group further divided into PBS, L929-Exo, and ADSC-Exo groups. RT-qPCR results showed that Atf3 expression levels in M2 microglia increased significantly after OGD/R (p < 0.01), and ADSC-Exo could reduce Atf3 expression (p < 0.05).

We further explored RNA-binding proteins (RBPs) in the ADSC-Exo that can regulate Atf3 expression. As shown in Fig. 3F, 101 RBPs that can bind to Atf3 were identified through the Starbase database. Second-generation sequencing revealed that 43 RBPs were related to the changes in Atf3 expression levels in microglia after MCAO (Fig. 3G). We further performed second-generation sequencing on L929-Exo and ADSC-Exo, and the results showed that a total of 3,651 genes were highly expressed in ADSC-Exo compared to L929-Exo (Fig. 3H). By intersecting Atf3 negatively correlated genes, RBPs regulating Atf3, and ADSC-Exo highly expressed genes, we obtained 5 candidate RBPs: Cstf2t, Upf1, Fxr2, Numa1, and Rbm10 (Fig. 3I). These 5 RBPs were abundant in ADSC-Exo, had reduced expression after MCAO, and could bind to Atf3. We divided primary M2 microglia into CON and OGD/R groups and detected mRNA level changes of the 5 candidate RBPs to determine the RBP regulating Atf3. The results showed that Fxr2 was the most significantly decreased RBP after OGD/R (p < 0.01, Fig. 3J). Western blot results further confirmed the presence of Fxr2 protein expression in both ADSC and ADSC-Exo (Fig. 3K). Actinomycin D experiments showed that after 6 h of ADSC-Exo intervention, the mRNA stability of Atf3 in M2 microglia was statistically different from the PBS group (p < 0.01, Fig. 3L), indicating that ADSC-Exo could reduce the stability of Atf3 mRNA and accelerate its degradation. We performed a RIP experiment to verify that the Fxr2 protein can bind to Atf3 mRNA. Detection of proteins in the complex showed that Fxr2 protein was detected in both the Input and IP group samples, while no Fxr2 protein was seen in the IgG group (Fig. 3M). Meanwhile, the detection of RNA in the complex showed that the content of Atf3 mRNA in the IP group was significantly higher than that in the IgG group (p < 0.001, Fig. 3N). The RIP experiment demonstrated that the Fxr2 protein can bind to Atf3 mRNA to form a protein-RNA complex.

### Regulation of the Fxr2/Atf3 axis reduced the ferroptosis sensitivity of M2 microglia

**Fig. 4:**
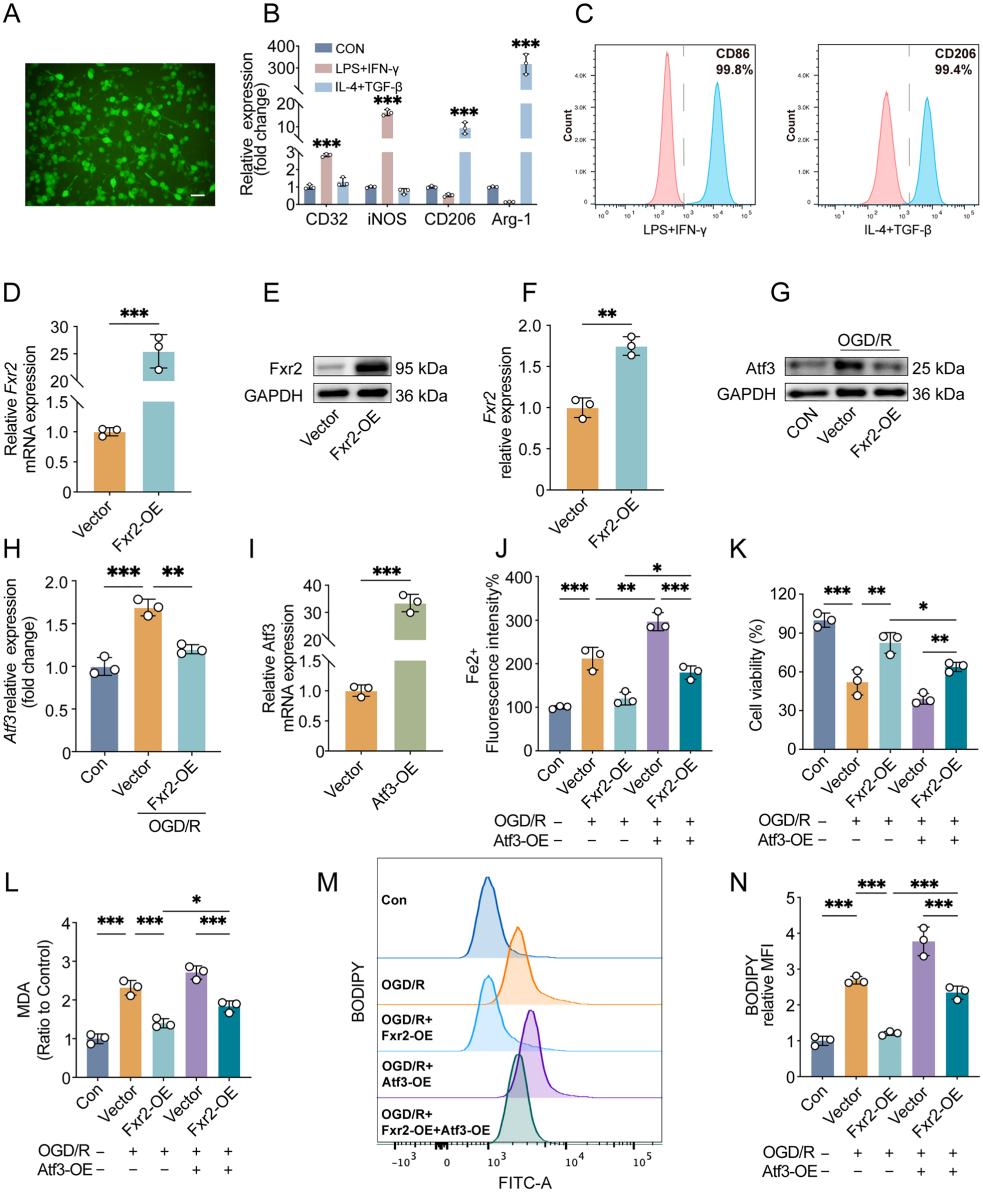
Regulation of the Fxr2/Atf3 axis reduced the ferroptosis sensitivity of M2 microglia. **A** Fluorescence microscopy assesses the infection efficiency of lentiviral vectors on BV2 microglia at MOI = 500; scale bar = 20 μm. **B** After induction with LPS (100 ng/mL) + IFN-γ (20 ng/mL) or IL-4 (20 ng/mL) + TGF-β (20 ng/mL) for 24 h, RT-qPCR was used to detect the expression levels of M1 and M2 subtype marker molecules in BV2 microglia under different interventions; n = 3. **C** Flow cytometry evaluates the proportions of M1 and M2 subtype marker molecules in BV2 microglia under different interventions; n = 3. **D** RT-qPCR assesses the overexpression effect of Fxr2 in microglia; n = 3. **E**, **F** Western blot evaluates the overexpression effect of Fxr2 in microglia; n = 3. **G**, **H** Western blot assesses the effect of Fxr2 overexpression on Atf3 expression levels in M2 microglia after OGD/R (3.5/24 h); n = 3. **I** RT-qPCR evaluates the overexpression effect of Atf3 in microglia; n = 3. **J** The ferrous iron level (Fe2 +) of M2 microglia in different groups was assessed by the FerroOrange probe, n = 3. **K** CCK-8 assay evaluates the changes in the cell survival rate of M2 microglia under different interventions; n = 3. **L** Malondialdehyde (MDA) assay evaluates the levels of lipid peroxidation products in M2 microglia under different interventions; n = 3. **M**, **N** C11-BODIPY 581/591 probe evaluates the levels of lipid ROS in M2 microglia under different interventions; n = 3. OE: overexpression; *p < 0.05; **p < 0.01; ***p < 0.001

To elucidate whether ADSC-Exo can regulate the ferroptosis of M2 microglia through the Fxr2/Atf3 axis, we first conducted a preliminary validation by modulating the expression of Fxr2 and Atf3 in M2 microglia. As shown in Fig. 4A, lentiviral vectors successfully transfected BV2 microglia (MOI = 500). On this basis, we constructed stable BV2 microglia lines overexpressing Fxr2 and further induced into M1 and M2 microglia. As shown in Fig. 4B, after induction with LPS and IFN-γ, the expression of M1 microglial markers CD32 and iNOS was significantly increased compared to the control group (p < 0.001), and after induction with IL-4 and TGF-β, the expression of M2 microglial markers CD206 and Arg-1 was significantly increased compared to the control group (p < 0.001). Flow cytometry analysis showed that the M1 microglial marker CD86 reached 99.8% after induction with LPS and IFN-γ, and the M2 microglial marker CD206 reached 99.4% after induction with IL-4 and TGF-β (Fig. 4C). These results indicate that we successfully induced the polarization of BV2 microglia lines toward M1 or M2 subtypes. In M2-type BV2 microglia, Fxr2 mRNA (p < 0.001, Fig. 4D) and protein (p < 0.01, Fig. 4E, F) expression levels in the Fxr2 overexpression group (Fxr2-OE group) were significantly higher than those in the control group (Vector group), indicating that we successfully constructed M2-type BV2 microglia with increased expression of Fxr2.

We divided M2-type BV2 microglia into the CON and OGD/R groups, with the OGD/R groups further divided into the Vector and Fxr2-OE groups. Western blot experiments demonstrated that Atf3 expression increased after OGD/R (p < 0.001), while Atf3 expression was reduced in the Fxr2-OE group compared to the Vector group (p < 0.01, Fig. 4G, H). This indicates that Fxr2 can regulate the expression of Atf3 in M2 microglia after OGD/R. Subsequently, we overexpressed Atf3 in BV2 microglia (Fig. 4I) to observe whether Fxr2's effect on cell ferroptosis was achieved by regulating Atf3 expression. FerroOrange probe detection showed that the intracellular Fe2 + level of M2-type BV2 microglia increased after OGD/R (p < 0.001), and overexpression of Fxr2 reduced Fe2 + level (p < 0.01). Still, the decrease in Fe2 + level was reversed due to overexpression of Atf3 (p < 0.05, Fig. 4J). CCK-8 assay results showed that the cell viability of M2-type BV2 microglia decreased after OGD/R (p < 0.001), while overexpression of Fxr2 increased cell viability (p < 0.01). However, the increased cell viability was reversed due to overexpression of Atf3 (p < 0.05, Fig. 4K). Similarly, MDA results indicated that the MDA content in M2-type BV2 microglia increased after OGD/R (p < 0.001), and overexpression of Fxr2 reduced MDA content (p < 0.001). Still, the decrease in MDA content was reversed due to overexpression of Atf3 (p < 0.05, Fig. 4L). C11-BODIPY probe detection showed that the lipid ROS levels in M2-type BV2 microglia increased after OGD/R (p < 0.001), and overexpression of Fxr2 reduced lipid ROS levels (p < 0.001). Still, the decrease in lipid ROS levels was reversed due to overexpression of Atf3 (p < 0. 001, Fig. 4M, N). The above experiments demonstrate that Fxr2 can inhibit the ferroptosis of M2-type microglia after OGD/R. Still, this protective effect can be weakened by overexpression of Atf3, indicating that Fxr2’s regulation of ferroptosis is directly related to Atf3.

### ADSC-Exo reduced ferroptosis sensitivity of M2 microglia through the Fxr2/Atf3/Slc7a11 axis

**Fig. 5:**
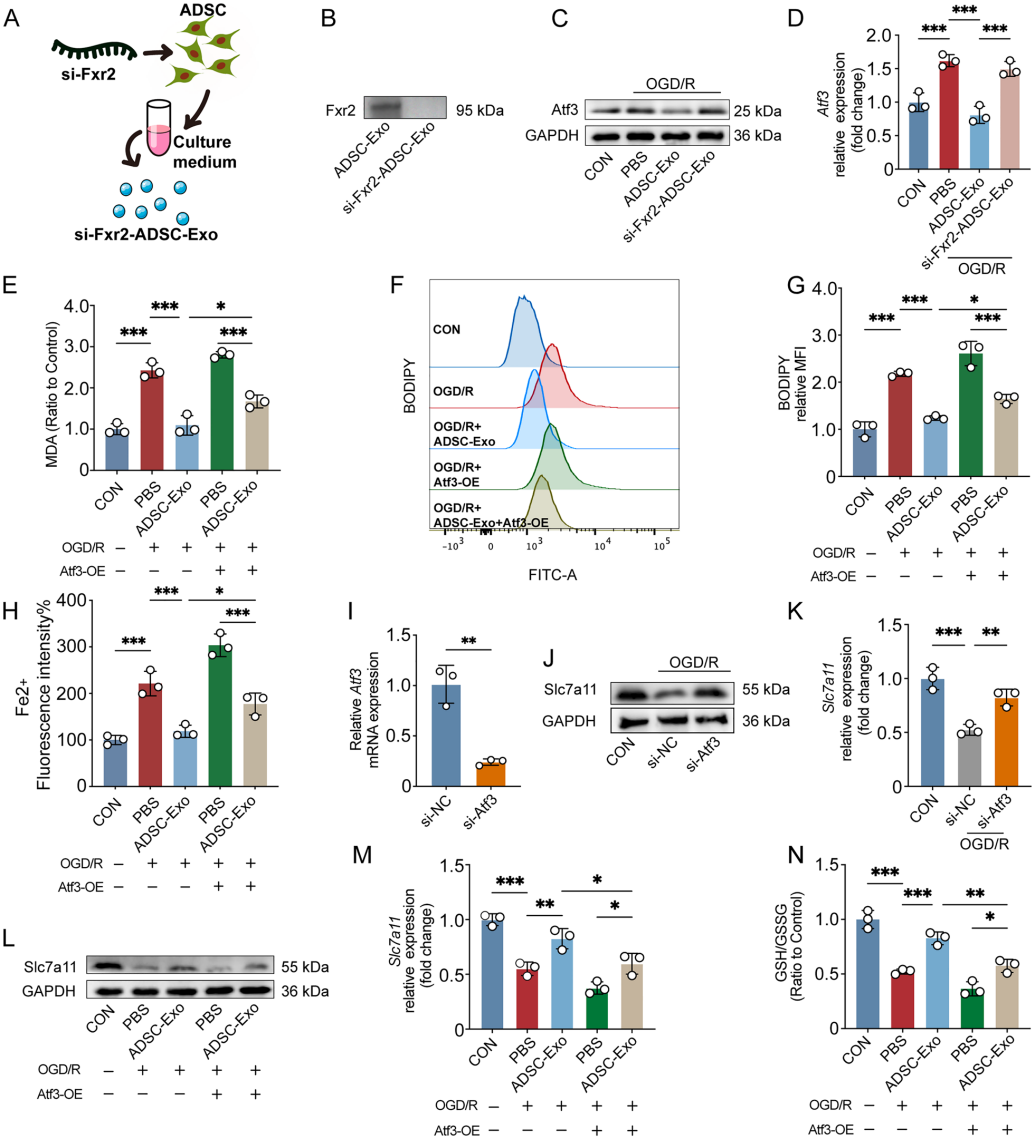
ADSC-Exo reduced ferroptosis sensitivity of M2 microglia through the Fxr2/Atf3/Slc7a11 axis.** A** Schematic illustration of the process for obtaining ADSC-Exo after knocking down Fxr2 in P4 ADSCs. **B** Western blot analysis of the effect of Fxr2 knockdown in ADSC-Exo. **C**, **D** Western blot assessment of the impact of ADSC-Exo (20 μg/mL, 24 h) and si-Fxr2-ADSC-Exo (20 μg/mL, 24 h) on Atf3 expression in M2 BV2 microglia after OGD/R, n = 3. **E** Malondialdehyde (MDA) evaluation of lipid peroxidation levels in different intervention groups of M2 microglia, n = 3. **F**, **G** C11-BODIPY 581/591 probe evaluates the levels of lipid ROS in M2 microglia under different interventions; n = 3. **H** The ferrous iron level (Fe_2_ +) of M2 microglia in different groups was assessed by the FerroOrange probe, n = 3. **I** RT-qPCR assessment of the effect of siRNA-mediated Atf3 knockdown, n = 3. **J**, **K** Western blot evaluation of changes in Slc7a11 expression in M2 BV2 microglia after Atf3 knockdown, n = 3. **L**, **M** Western blot assessment of changes in Slc7a11 expression in M2 BV2 microglia after OGD/R in different intervention groups, n = 3. **N** Glutathione detection for assessing GSH/GSSG ratio in M2 BV2 microglia after OGD/R in different intervention groups, n = 3. *p < 0.05; **p < 0.01; ***p < 0.001

To clarify whether ADSC-Exo can regulate Atf3 expression in M2 microglia through its cargo Fxr2, thus affecting cell ferroptosis, and further explore the molecular mechanism of the Fxr2/Atf3 axis on ferroptosis, we conducted further investigations. As shown in Fig. 5A, we knocked down Fxr2 in ADSCs using siRNA and collected the secreted exosomes si-Fxr2-ADSC-Exo. Western blot showed that after knocking down Fxr2 in ADSCs, the protein content of Fxr2 in si-Fxr2-ADSC-Exo was significantly reduced (Fig. 5B). Subsequently, we observed whether ADSC-Exo administration could reduce Atf3 expression in M2 microglia. As shown in Fig. 5C, D, compared with PBS, ADSC-Exo could reduce the expression of Atf3 in M2-type BV2 microglia after OGD/R (p < 0.001), but si-Fxr2-ADSC-Exo could not downregulate Atf3 expression (p > 0.05). This indicates that ADSC-Exo regulates Atf3 expression in M2-type microglia through its cargo Fxr2. As shown in Fig. 5E, ADSC-Exo could reduce the MDA content in M2-type BV2 microglia after OGD/R (p < 0.001), while overexpression of Atf3 could prevent ADSC-Exo from reducing the MDA content (p < 0.05). Similarly, C11-BODIPY probe detection showed that ADSC-Exo could reduce the lipid ROS levels in M2-type BV2 microglia after OGD/R (p < 0.001). Still, overexpression of Atf3 could prevent ADSC-Exo from reducing the lipid ROS levels (p < 0.05, Fig. 5F, G). FerroOrange probe detection showed that ADSC-Exo could reduce the intracellular Fe2 + level of M2-type BV2 microglia after OGD/R (p < 0.001), while overexpression of Atf3 could prevent ADSC-Exo from reducing the Fe2 + level (p < 0.05, Fig. 5H). These suggest that Fxr2 in ADSC-Exo also regulates cell ferroptosis through Atf3.

Based on previous reports [40], we believe that Atf3's pro-ferroptosis function may be related to Slc7a11. Therefore, we further explored whether ADSC-Exo affects M2-type microglia ferroptosis through the Fxr2/Atf3/Slc7a11 axis. We knocked down Atf3 in BV2 microglia using siRNA, and RT-qPCR showed that Atf3 expression was significantly reduced (p < 0.01, Fig. 5I). Western blot results showed that Slc7a11 expression decreased after OGD/R (p < 0.001), and Slc7a11 expression increased after knocking down Atf3, indicating that Atf3 could regulate Slc7a11 expression (p < 0.01, Fig. 5J, K). We further found that ADSC-Exo could increase the expression level of Slc7a11 after OGD/R (p < 0.01), but overexpression of Atf3 would weaken the effect of ADSC-Exo on Slc7a11 expression (p < 0.05, Fig. 5L, M). At the same time, glutathione detection found that the GSH/GSSG ratio decreased after OGD/R (p < 0.001), and ADSC-Exo could increase the GSH/GSSG ratio after OGD/R (p < 0.001). Still, overexpression of Atf3 would weaken the effect of ADSC-Exo on the GSH/GSSG ratio (p < 0.01, Fig. 5N). These results suggest that ADSC-Exo can affect the glutathione content and regulate M2 microglia ferroptosis sensitivity through the Fxr2/Atf3/Slc7a11 axis.

### Construction of engineered ADSC-Exo targeting M2 microglia

**Fig. 6:**
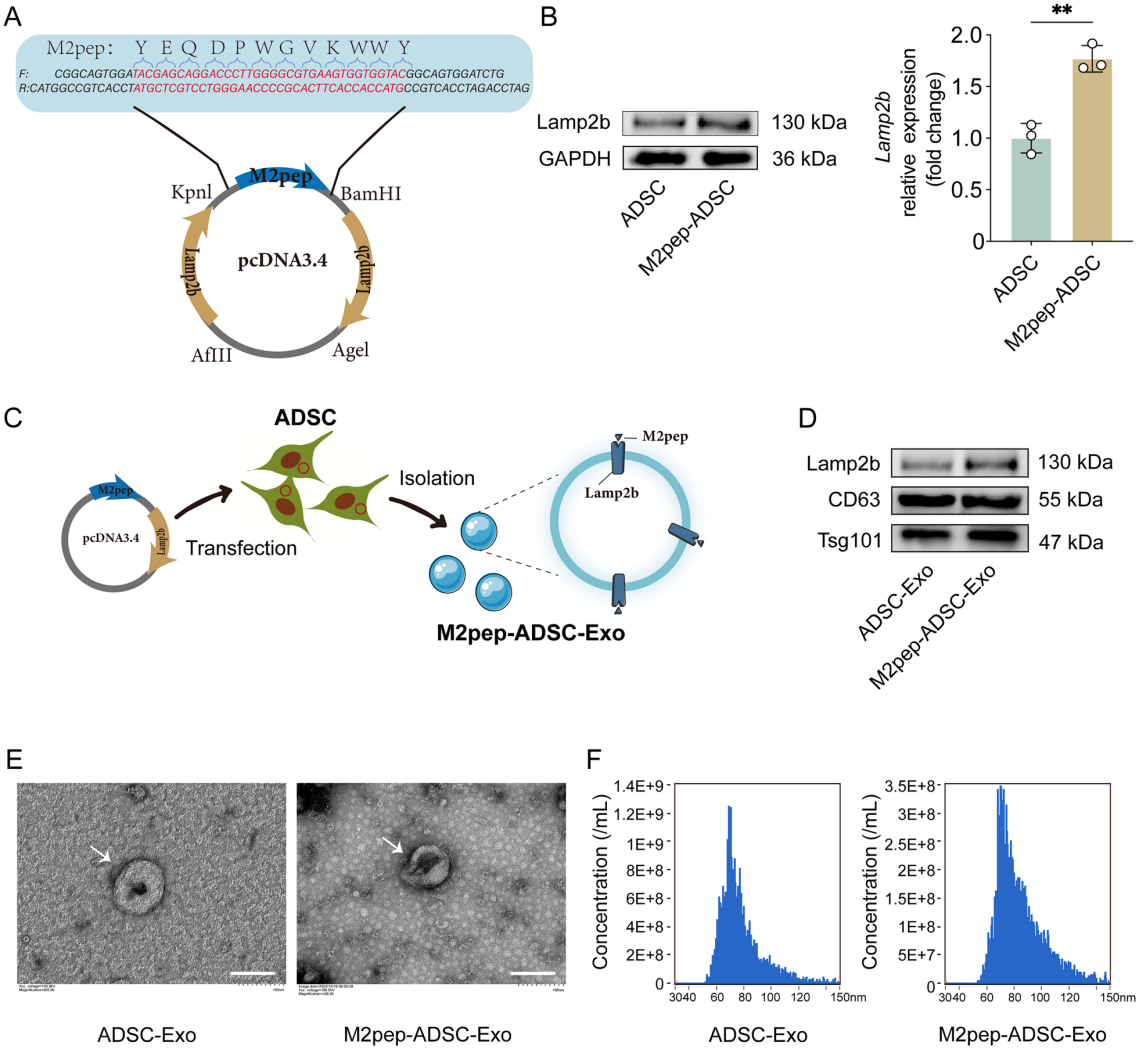
Construction of M2pep-ADSC-Exo targeting M2 Microglia. **A** Schematic representation of the M2pep-Lamp2b recombinant plasmid. **B** Western blot analysis of Lamp2b protein expression in ADSCs following transfection with the M2pep-Lamp2b recombinant plasmid, n = 3. **C** Flowchart depicting the process of obtaining M2pep-ADSC-Exo. **D** Western blot assessment of exosomal marker proteins and Lamp2b expression levels in ADSC-Exo and M2pep-ADSC-Exo. **E** Transmission electron microscopy observation of the morphology of ADSC-Exo and M2pep-ADSC-Exo, with white arrows indicating exosomes, scale bar = 100 nm. **F** NanoFCM measurement of the particle size range of ADSC-Exo and M2pep-ADSC-Exo. **p < 0.01

To further enhance the efficacy of ADSC-Exo, we performed engineered modifications to improve its targeting capabilities. First, we constructed a Lamp2b-M2pep recombinant plasmid (Fig. 6A) and transfected it into ADSCs. Western blot analysis revealed a significant increase of Lamp2b expression in M2pep-ADSCs compared to natural ADSCs (p < 0.01), indicating the successful transfection of the Lamp2b-M2pep recombinant plasmid into ADSCs (Fig. 6B). The exosomes secreted by M2pep-ADSCs, M2pep-ADSC-Exo, were collected using ultracentrifugation and subsequently characterized (Fig. 6C). As shown in Fig. 6D, both ADSC-Exo and M2pep-ADSC-Exo expressed exosome markers CD63 and Tsg101, with a higher Lamp2b expression in M2pep-ADSC-Exo compared to ADSC-Exo. The transmission electron microscope revealed that ADSC-Exo and M2pep-ADSC-Exo exhibited a typical cup-shaped morphology with a round, concave center (Fig. 6E). Nanoparticle size analysis showed that the average diameter of ADSC-Exo and M2pep-ADSC-Exo was 74.2 ± 15.0 nm and 79.8 ± 16.4 nm, respectively (Fig. 6F). These experiments indicate that we have successfully constructed ADSC-Exo carrying the M2pep targeting peptide and that M2pep-ADSC-Exo meets exosome characterization criteria.

### M2pep-ADSC-Exo exhibits significant targeting specificity for M2 microglia, further inhibiting M2 microglia ferroptosis and improving inflammation

**Fig. 7:**
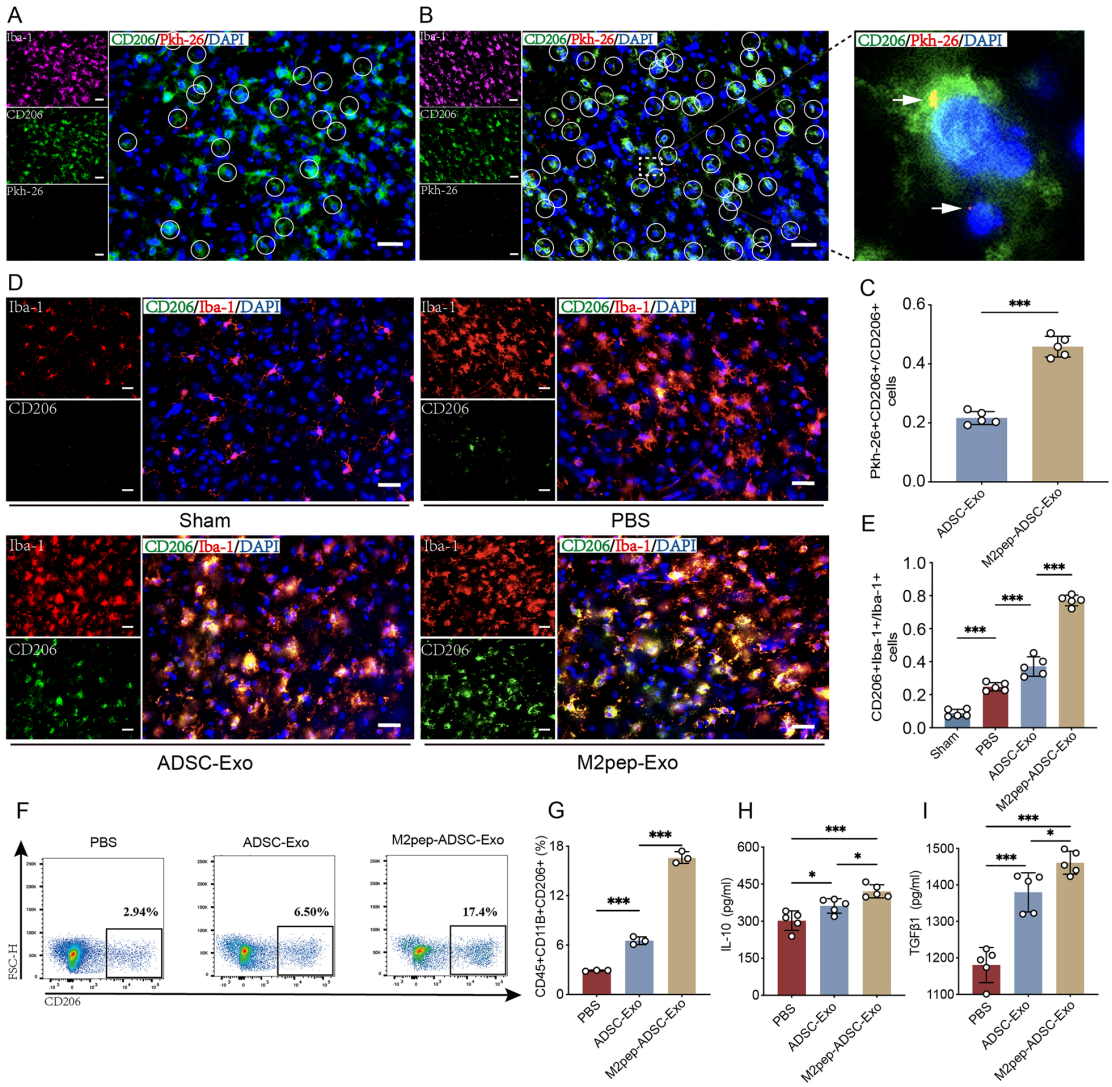
M2pep-ADSC-Exo exhibits significant targeting specificity for M2 microglia, further inhibiting M2 microglia ferroptosis and improving inflammation. Intranasal administration of PKH-26-labeled **A** ADSC-Exo (1000 μg/mL, 10 μL) or **B** M2pep-ADSC-Exo (1000 μg/mL, 10 μL) to MCAO mice, followed by immunofluorescence examination of the ischemic penumbra brain tissue 6 h later, assessing the uptake capability of M2 microglia (green) for ADSC-Exo or M2pep-ADSC-Exo (red). White circles indicate internalized ADSC-Exo or M2pep-ADSC-Exo. The white arrows indicate internalized M2pep-ADSC-Exo. Scale bar = 20 μm, n = 5. **C** Quantitative analysis of the proportion of M2 microglia (PKH-26 + CD206 + cells) that have taken up ADSC-Exo or M2pep-ADSC-Exo relative to the total M2 microglia population (CD206 + cells). n = 5. **D**, **E** Immunofluorescence assessment of the proportion of M2 microglia in the cortical ischemic penumbra brain tissue of mice in the PBS (10 μL, 3 days), ADSC-Exo (1000 μg/mL, 10 μL/day, 3 days), and M2pep-ADSC-Exo (1000 μg/mL, 10 μL/day, 3 days) intervention groups 3 days after MCAO. Scale bar = 20 μm, n = 5. **F**, **G** Flow cytometry assessment of the proportion of M2 microglia in the cortical brain tissue of mice 3 days after MCAO, treated with PBS (10 μL, 3 days), ADSC-Exo (1000 μg/mL, 10 μL/day, 3 days), and M2pep-ADSC-Exo (1000 μg/mL, 10 μL/day, 3 days); n = 3. **H** Cytokine array analysis of IL-10 and **I** TGFβ1 levels in cortical brain tissue 3 days after MCAO in mice, n = 5. *p < 0.05; **p < 0.01; ***p < 0.001

To verify the targeting capability of M2pep-ADSC-Exo for M2 microglia, we induced primary microglia into M1 and M2 phenotypes in vitro and incubated them with PKH-26-labeled ADSC-Exo or M2pep-ADSC-Exo for 1 h. Fluorescence microscopy observations revealed no difference in the uptake rate of ADSC-Exo between M1 and M2 microglia. However, there was a significant difference in the uptake rate of M2pep-ADSC-Exo, with M2 microglia exhibiting a higher uptake rate than M1 microglia (p < 0.001, Supplementary Fig. 5A, B). Furthermore, the uptake rate of M2pep-ADSC-Exo by M1 microglia was lower than that of ADSC-Exo (p < 0.01). In comparison, the uptake rate of M2pep-ADSC-Exo by M2 microglia was higher than that of ADSC-Exo (p < 0.01, Supplementary Fig. 5A, B). To further validate the ability of M2pep-ADSC-Exo to target M2 microglia in vivo, we administered PKH-26-labeled ADSC-Exo or M2pep-ADSC-Exo to MCAO mice via intranasal delivery immediately after modeling. The mice were sacrificed 6 h after administration. Immunofluorescence staining revealed that, compared to the ADSC-Exo group, mice treated with M2pep-ADSC-Exo exhibited higher PKH-26 + CD206 + signals (p < 0.001, Fig. 7A–C). These findings indicate that the constructed M2pep-ADSC-Exo shows targeting specificity for M2 microglia and can selectively enter M2 microglia with high efficiency.

We further investigated the regulatory effects of M2pep-ADSC-Exo on the ferroptosis of M2 microglia and its impact on the inflammatory microenvironment in vivo. We divided the mice into sham and model groups and subdivided the model group into PBS intervention, ADSC-Exo intervention, and M2pep-ADSC-Exo intervention groups. Three days after administration, observation of the cortical ischemic penumbra revealed that ADSC-Exo could increase the number of M2 microglia during the acute phase (p < 0.001). At the same time, M2pep-ADSC-Exo could further enhance the quantity of M2 microglia (p < 0.001, Fig. 7D, E). As shown in the representative flow cytometry gating plot (Supplementary Fig. 6), CD45^+^CD11b^+^ microglia were selected, followed by further identification of CD206^+^ M2 microglia. The results demonstrated that the proportion of M2 microglia was increased in the ADSC-Exo group compared to the PBS group after MCAO (p < 0.001). Moreover, M2pep-ADSC-Exo further enhanced the proportion of M2 microglia (p < 0.001, Fig. 7F, G). These experiments indicate that M2pep-ADSC-Exo exhibits a superior effect on M2 microglia, suppressing M2 microglia ferroptosis and mitigating the decrease in the quantity of M2 microglia during the acute phase. Additionally, analysis of inflammatory factors revealed elevated levels of IL-10 (p < 0.05) and TGF-β (p < 0.001) in the ischemic penumbra cortical brain tissue of ADSC-Exo group mice compared to the PBS group after MCAO. Notably, M2pep-ADSC-Exo further increased IL-10 (p < 0.001) and TGF-β levels (p < 0.001), suggesting an enhanced modulation of the inflammatory response (Fig. 7H, I).

### *M2pep-ADSC-Exo further inhibits ferroptosis and improves neurological function *in vivo

**Fig. 8:**
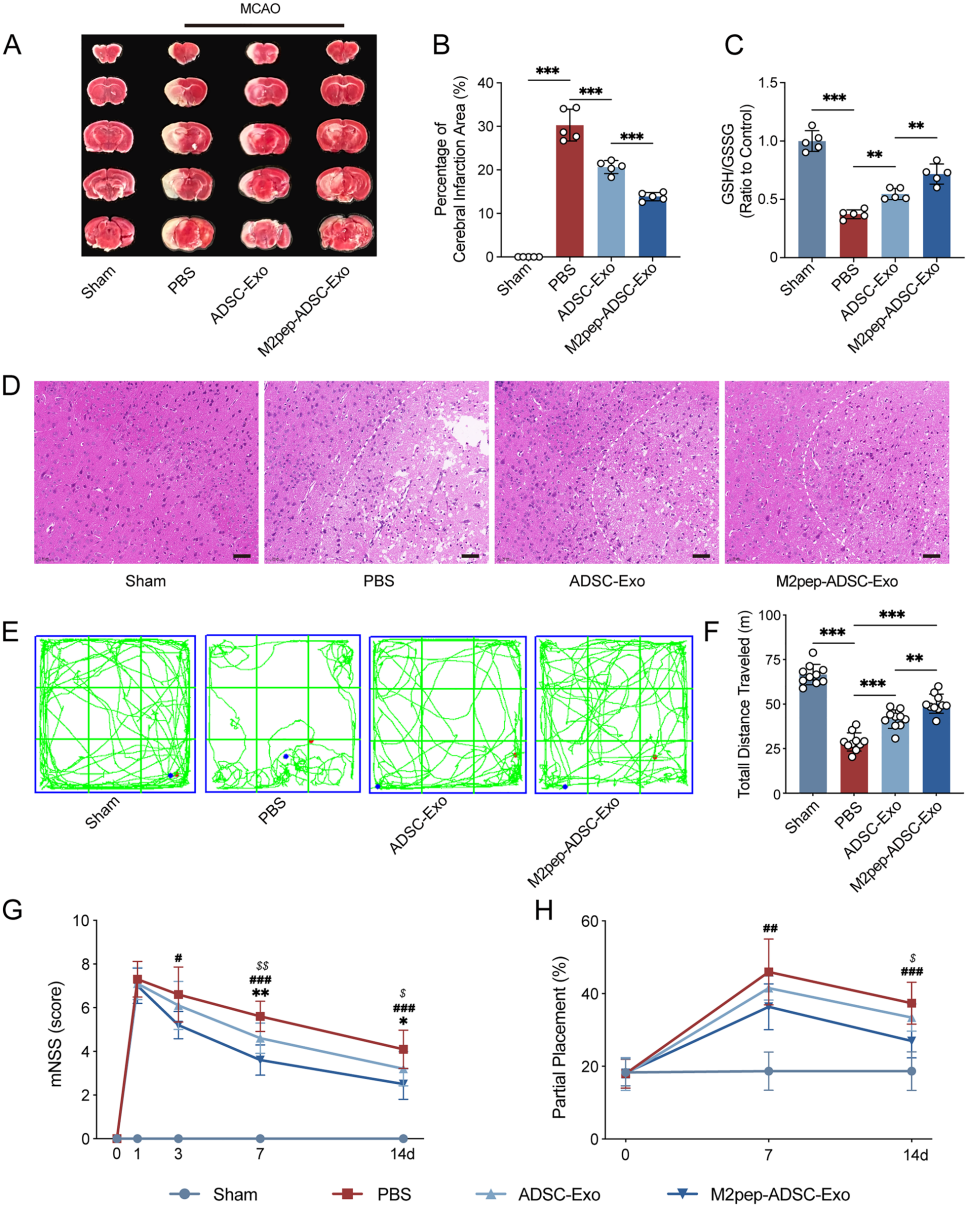
M2pep-ADSC-Exo further Inhibit Ferroptosis and improve Neurological function in vivo. **A**, **B** TTC staining was used to assess the effect of M2pep-ADSC-Exo (1000 μg/mL, 5 μL/day, 3 days) or ADSC-Exo (1000 μg/mL, 5 μL/day, 3 days) on the brain infarct size of mice 3 days after MCAO. n = 5. **C** Glutathione assay was used to assess the effect of M2pep-ADSC-Exo (1000 μg/mL, 5 μL/day, 3 days) or ADSC-Exo (1000 μg/mL, 5 μL/day, 3 days) on the GSH/GSSG ratio in the cortical brain tissue of mice 3 days after MCAO; n = 5. **D** HE staining was employed to evaluate the effects of M2pep-ADSC-Exo (1000 μg/mL, 5 μL/day, 3days) or ADSC-Exo (1000 μg/mL, 5 μL/day, 3days) on the amelioration of cellular damage in the cortical brain tissue of the ischemic penumbra on the infarcted side. The white dashed line indicates the boundary of the ischemic penumbra. Scale bar = 50 μm. **E**, **F** The motor function of mice was assessed by open-field test at 3 days after the MCAO; n = 10. **G** mNSS scoring assessment of the improvement in neurological function deficits in mice at 1, 3, 7, and 14 days after MCAO by the intervention of M2pep-ADSC-Exo (1000 μg/mL, 5 μL/day, 3 days) or ADSC-Exo (1000 μg/mL, 5 μL/day, 3 days); n = 10. *p < 0.05; **p < 0.01; ***p < 0.001

TTC staining revealed a pronounced cerebral infarction in brain tissue after modeling. ADSC-Exo was able to reduce the infarct area significantly (p < 0.001), and M2pep-ADSC-Exo further reduced the infarct area (p < 0.001, Fig. 8A, B). GSH measurements revealed a decreased GSH/GSSG ratio after MCAO (p < 0.001). ADSC-Exo increased the GSH/GSSG ratio after MCAO (p < 0.01), while M2pep-ADSC-Exo further elevated the GSH/GSSG ratio (p < 0.01, Fig. 8C). The results of HE staining revealed neuronal degeneration and necrosis in the infarcted cortical region, characterized by nuclear condensation, increased staining intensity, unclear structural integrity, accompanied by cytoplasmic acidophilia, and some neurons exhibited swelling and rupture with disorganized cellular arrangements. ADSC-Exo alleviated brain tissue damage, while M2pep-ADSC-Exo further mitigated brain tissue injury (Fig. 8D). Open-field test results demonstrated a significant decline in motor function after MCAO in mice (p < 0.001). ADSC-Exo improved motor function (p < 0.001), and M2pep-ADSC-Exo further improved motor function (p < 0.01, Fig. 8E, F). The mNSS showed that the neurological deficits of mice in the ADSC-Exo group were significantly less than that in the PBS group at 7 days (p < 0.01) and 14 days (p < 0.05) after MCAO. M2pep-ADSC-Exo intervention, when compared to PBS intervention, demonstrated statistically significant differences at 3d (p < 0.05), 7 days (p < 0.001), and 14 days (p < 0.001), and exhibited milder neurological deficit at 7 days (p < 0.01) and 14 days (p < 0.05) compared to ADSC-Exo intervention (Fig. 8G). The foot fault test showed no statistically significant difference in the partial placement rate between the ADSC-Exo and the PBS groups at 7d and 14d after MCAO (p > 0.05). M2pep-ADSC-Exo intervention, in comparison to PBS intervention, exhibited statistically significant differences at 7 days (p < 0.01) and 14 days (p < 0.001) and displayed a reduced partial placement rate at 14 days (p < 0.05) compared to ADSC-Exo intervention (Fig. 8G).

## Discussion

As the first line of immune defense in the brain, microglia play a crucial role in stroke progression. Our previous study demonstrated significant ferroptosis in brain cells following IS. However, the focus was primarily on neurons, and further investigation into the ferroptosis status of microglia was not conducted [30]. In this study, we first investigated whether microglia undergo ferroptosis after stroke. We isolated adult mouse brain tissue and used CD11b MicroBeads to sort microglia. TEM and Lipid ROS probes indicate that microglia undergo significant ferroptosis after IS, and our choice of using microglia from adult mouse models for experiments provides more persuasive evidence than in vitro cell experiments.

On this basis, we attempted to observe the intervention effect of ferroptosis inhibitor Fer-1 and ADSC-Exo. The results showed that the impact of Fer-1 and ADSC-Exo were different. Inflammation factor detection indicated that ADSC-Exo had a regulatory effect on pro-inflammatory and anti-inflammatory factors, while Fer-1 only had a regulatory effect on anti-inflammatory factors. Studies have shown that microglia are rapidly activated and polarized into different subtypes after IS, with pro-inflammatory M1 microglia secreting large amounts of pro-inflammatory factors such as TNFα, IL-1β, and iNOS, while anti-inflammatory M2 microglia secrete large quantities of anti-inflammatory factors such as TGF-β and IL-10 [41–43]. As a specific inhibitor of ferroptosis, Fer-1 can only affect the inflammation factor levels related to M2 microglia. We speculate that the degree of ferroptosis in M2 microglia is greater after IS; therefore, the rescue effect of Fer-1 is relatively more pronounced. A study has shown that M2 macrophages or microglia are more sensitive to ferroptosis inducers, while M1 macrophages or microglia have low sensitivity [21]. Therefore, we hypothesize that there is also a difference in ferroptosis sensitivity between different subtypes of microglia during IS.

To test this hypothesis further, we extracted primary microglia from neonatal mice and induced their differentiation into M1 and M2 phenotypes. We observed the ferroptosis of different subtypes of microglia under the conditions of ferroptosis inducer RSL3 and OGD/R. The results showed that RSL3 and OGD/R could cause significant ferroptosis in M2 microglia, while M1 microglia had a higher resistance. This suggests that M2 microglia are highly sensitive to ferroptosis, and this characteristic persists under IS conditions.

We further investigated the regulatory role of ADSC-Exo on the sensitivity of M2 microglia to ferroptosis. We first determined the optimal ADSC-Exo dosage for intervention in M2 microglia to be 20 μg/mL. Cell viability, lipid ROS, and lipid peroxidation product MDA assays demonstrated that ADSC-Exo could inhibit ferroptosis in M2 microglia induced by RSL-3 or OGD/R. Concurrently, ELISA experiments revealed that ADSC-Exo could modulate the secretion of inflammatory factors in microglia caused by OGD/R, characterized by a decrease in the pro-inflammatory factor TNFα and an increase in the anti-inflammatory factor IL-10. Through conditional medium experiments, we found that the inhibitory effect of ADSC-Exo on the inflammatory microenvironment of microglia further influenced neuronal survival. Previous research has shown that microglia and neurons interact, and the inflammatory microenvironment constructed by microglia significantly affects neuronal survival [44], which is consistent with our conclusions.

After determining the regulatory role of ADSC-Exo on ferroptosis in M2 microglia, we further explored its underlying molecular mechanism. Since it is impossible to directly isolate a sufficient quantity of M2 microglia from adult mouse brains, we isolated cortical brain tissue from sham-operated and MCAO mice and then used CD11b MicroBeads to isolate microglia for second-generation sequencing. Sequencing analysis showed that the expression of the ferroptosis-related gene Atf3 in MCAO mouse microglia was significantly increased. In vivo experiments confirmed that Atf3 increased after stroke, and in vitro experiments further demonstrated that Atf3 was elevated considerably in M2 microglia. ADSC-Exo could reduce Atf3 expression levels, while the control exosomes L929-Exo could not. The feasibility of using fibroblast-derived exosomes as control exosomes for ADSC-Exo has been demonstrated in previous studies [45]. Therefore, we believe that Atf3 may be the critical gene promoting ferroptosis in M2 microglia, and ADSC-Exo regulates Atf3 expression through its cargo. Atf3, activating transcription factor 3, is a common stress sensor involved in the complex process of cellular stress responses. Studies have shown that Atf3 can inhibit System Xc-, deplete intracellular GSH, and thus promote ferroptosis [40]. Another study showed that Atf3 could promote glioma cell ferroptosis by increasing iron and H_2_O_2_ content [46]. These studies indicate that Atf3 plays a crucial role in promoting cell ferroptosis.

To clarify which cargo of ADSC-Exo regulates Atf3 expression, we screened RBPs that can bind to Atf3. We found that the expression of multiple RBPs changed after MCAO, and among them, 43 RBPs were correlated with Atf3. To further determine whether ADSC-Exo contains RBPs associated with Atf3, we collected ADSC-Exo and control group exosomes L929-Exo for sequencing. A Venn diagram revealed 5 RBPs intersections, which were highly expressed in ADSC-Exo, downregulated after MCAO and correlated with Atf3. Since the sequencing target was microglia rather than M2 microglia, we further verified the expression changes of the 5 RBPs in primary M2 microglia, and the results showed that Fxr2 had the most significant change. Fxr2, also known as RNA-binding protein Fxr2, is a synthetically producible protein that offers possibilities for clinical translation. Previous studies have shown that Fxr2 is involved in numerous neurological diseases [47–49]. Fxr2 participates in regulating gene expression changes and post-transcriptional processes, playing an essential role in neurogenesis and the regulation of adult neural stem cell self-renewal and maintenance [50, 51]. Through actinomycin D and RIP experiments, we confirmed that Fxr2 could bind to Atf3 and reduce Atf3 mRNA stability.

RBPs are important regulatory factors in RNA metabolism, with RBP-RNA interactions ranging from single protein-RNA component interactions to the assembly of multiple RBPs and RNA molecules (such as spliceosomes) [52]. They are crucial in all steps of gene expression cascades [53]. The role of RBP expression differences in human diseases is an essential area of research, and the potential application of RBPs as therapeutic targets or diagnostic biomarkers is a rapidly developing research field. RBPs have been widely used in preclinical research of IS [54]. ADSC-Exo also contains a large number of RBPs, but the molecular functions of these RBPs have not been thoroughly studied. Therefore, our study on RBPs is of great significance.

To further confirm the critical role of the Fxr2/Atf3 axis in the ferroptosis of M2 microglia, we constructed an Fxr2-overexpressing BV2 stable cell line. We found that Fxr2 overexpression could reduce Atf3 expression levels after OGD/R and then inhibit M2 microglia ferroptosis, and this effect was weakened by Atf3 overexpression. This suggests that the Fxr2/Atf3 axis plays a vital role in M2 microglia ferroptosis and provides a basis for exploring the regulation of M2 microglia ferroptosis by ADSC-Exo. We further observed the effects of ADSC-Exo on Atf3 expression levels and ferroptosis of M2 microglia by knocking down Fxr2 in ADSCs. ADSC-Exo could reduce Atf3 expression, while si-Fxr2-ADSC-Exo did not affect Atf3 expression. Furthermore, we found that ADSC-Exo regulated Atf3 by acting Slc7a11 to exert its anti-ferroptosis effect. Slc7a11 (solute carrier family 7 member 11, also known as xCT) plays a vital role in cellular defense against ferroptosis [55]. Slc7a11 imports extracellular cystine into cells, which is then converted into cysteine, promoting GSH synthesis and resisting ferroptosis through antioxidant effects [56].

Due to the requirement of ADSC-Exo to target M2 microglia for effective action, we must enhance the targeting ability of ADSC-Exo towards M2 microglia. We successfully constructed a Lamp2b-M2pep recombinant plasmid and transfected it into ADSCs to collect ADSC-Exo. Western blot demonstrated that the modified ADSC-Exo exhibited high expression of Lamp2b, indicating that we successfully loaded M2pep targeting peptide onto ADSC-Exo. We also characterized the modified ADSC-Exo using TEM, nanoparticle size measurements, and surface marker protein detection, showing that the modified ADSC-Exo met the identification criteria for exosomes [57]. M2 macrophage-targeting peptide (M2pep, YEQDPWGVKWWY) is a peptide capable of targeting M2 macrophages or microglia and is commonly used in cancer research [58]. Previous studies have shown that intravenous injection of M2pep leads to substantial accumulation in tumor-associated M2 macrophages in vivo [59]. We induced primary microglia into M1 and M2 phenotypes in vitro and treated them with ADSC-Exo and M2pep-ADSC-Exo, respectively. We found that M2 microglia exhibited a significantly higher uptake rate of M2pep-ADSC-Exo compared to ADSC-Exo, and M2 microglia also showed a higher uptake rate of M2pep-ADSC-Exo compared to M1 microglia. This indicates that M2pep-ADSC-Exo has significant targeting specificity for M2 microglia.

To further observe the in vivo effectiveness of M2pep-ADSC-Exo, we administered PKH-26-labeled ADSC-Exo or M2pep-ADSC-Exo to mice immediately after surgery. Immunofluorescence analysis revealed that M2pep-ADSC-Exo exhibited a more efficient targeting of M2 microglia. Then, we administered PBS, ADSC-Exo, and M2pep-ADSC-Exo to MCAO mice, respectively. Three days after MCAO, we isolated cortical brain tissue from the mice and performed immunofluorescence and flow cytometry. We found that the proportion of M2 microglia was lower in the PBS group, increased in the ADSC-Exo group, and further increased in the M2pep-ADSC-Exo group. This indicates that M2pep-ADSC-Exo can target M2 microglia in vivo and maintain their numbers by inhibiting microglia ferroptosis through the functionality of ADSC-Exo cargo. By assessing the levels of inflammatory factors in brain tissue, we observed that the M2pep-ADSC-Exo exhibited a superior capacity to alleviate the inflammatory microenvironment. Additionally, our findings revealed that M2pep-ADSC-Exo led to a pronounced improvement in the infarct area, the GSH/GSSG ratio within the cortical brain tissue, and the level of cellular injury in the ischemic penumbra. Moreover, our behavioral experiments revealed that M2pep-ADSC-Exo further promoted neurological function recovery and motor function.

This study indicated that diverse subtypes of microglia exhibited differential sensitivity to ferroptosis within the microenvironment of cerebral I/R injury for the first time. We clarified the effects and mechanisms of ADSC-Exo in reducing the ferroptosis sensitivity of M2 microglia. Several previous studies have reported the effect of ADSC-Exo on M1 microglia. Hu et al. found that ADSC-Exo could decrease M1-related markers and increase M2-related markers of primary microglia under LPS or OGD induction, promoting microglia M2 polarization [20]. Other research in brain injury and Alzheimer's disease models also indicated that ADSC-Exo could inhibit microglia M1 polarization while promoting microglia M2 polarization [60, 61]. These studies intervened with resting microglia and observed changes in the M1 and M2 microglia proportion, thus giving equal attention to M1 and M2 microglia. Yet, microglial polarization occurs rapidly after stroke; many microglia already polarize into the M2 phenotype shortly after successful reperfusion therapy. Then, their numbers rapidly decline in the subsequent days. Therefore, protecting existing M2 microglia and maintaining a high level of M2 microglia are crucial for suppressing the inflammatory microenvironment and alleviating reperfusion injury, holding significant clinical implications. Additionally, this study found that ADSC-Exo exert their effects through their cargo, Fxr2. Although ADSCs possess promising clinical translational potential due to their wide availability, convenience of sourcing, and high yield of exosomes, there is currently no evidence to suggest that Fxr2 is an exclusive protein component of ADSCs. Therefore, the mechanism uncovered in this study may have broad applicability, suggesting that exosomes derived from other types of MSCs may also possess similar effects, which requires further investigation.

## Conclusion

The present study found that microglia undergo ferroptosis after IS, and different subtypes of microglia exhibit varying sensitivities to ferroptosis, with M2 microglia being highly sensitive. The molecular mechanism is related to the increased expression of Atf3 in M2 microglia, and Fxr2 can bind to Atf3 mRNA, reducing its stability and thus inhibiting M2 microglia ferroptosis. At the same time, ADSC-Exo contains abundant Fxr2, and exogenous administration of ADSC-Exo can inhibit M2 microglia ferroptosis, suppress the inflammatory microenvironment, and ultimately affect neuronal survival through the Fxr2/Atf3/Slc7a11 pathway. Additionally, we successfully engineered ADSC-Exo to create M2pep-ADSC-Exo, which can target M2 microglia. Both in vitro and in vivo experiments demonstrated that M2pep-ADSC-Exo could target M2 microglia, increase the proportion of M2 microglia after IS, and enhance anti-ferroptosis capacity.

### Supplementary Information

Supplementary Material 1.

## Data Availability

The datasets used and/or analyzed during the current study are available from the corresponding author upon reasonable request.
