# Supplementary material for: Anti-ferroptosis exosomes engineered for targeting M2 microglia to improve neurological function in ischemic stroke
Source: J Nanobiotechnology. 2024 May 27;22:291. doi: 10.1186/s12951-024-02560-y (PMC11129432; doi:10.1186/s12951-024-02560-y)
Supplement: Supplementary file 1 — Supplementary Material 1. [file 12951_2024_2560_MOESM1_ESM.docx]

**
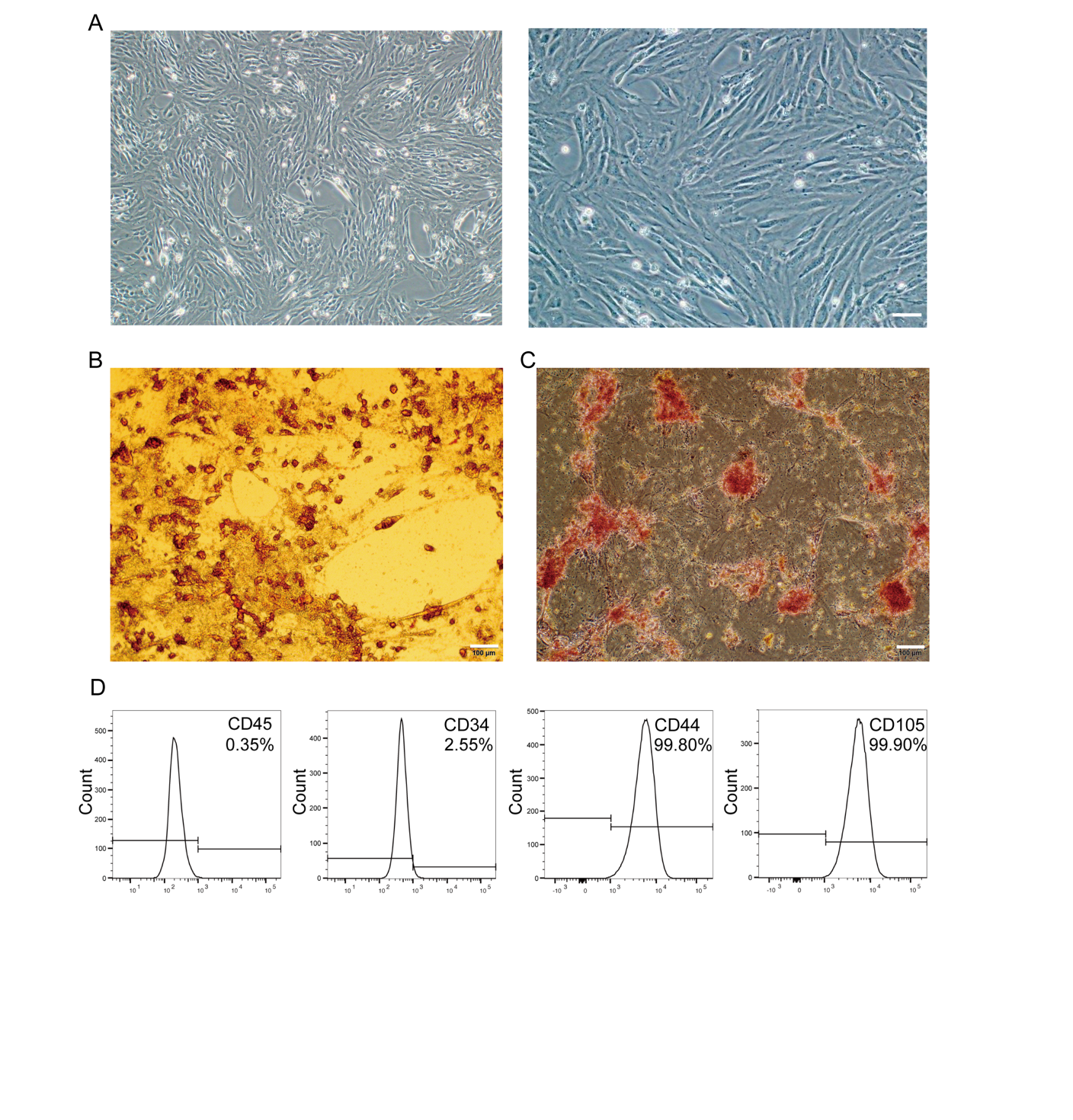
Supporting Information**

**Supplementary Figure 1:** **Isolation and identification of primary ADSCs.** **(A)** Morphological characteristics of ADSCs under a light microscope, scale = 20 μm. **(B)** P1 generation ADSCs after 7 days of adipogenic induction, showing numerous lipid droplets stained red with Oil Red O in the cytoplasm (white arrows), scale = 100 μm. **(C)** Alizarin Red S staining of P1 generation ADSCs after 14 days of osteogenic differentiation, demonstrating the presence of red calcium nodules (white arrows), scale = 100 μm. **(D)** Flow cytometric analysis shows that ADSCs surface markers CD45 and CD34 were negative expressions, while CD44 and CD105 were positive expressions, which meet the criteria for identifying ADSCs.

**
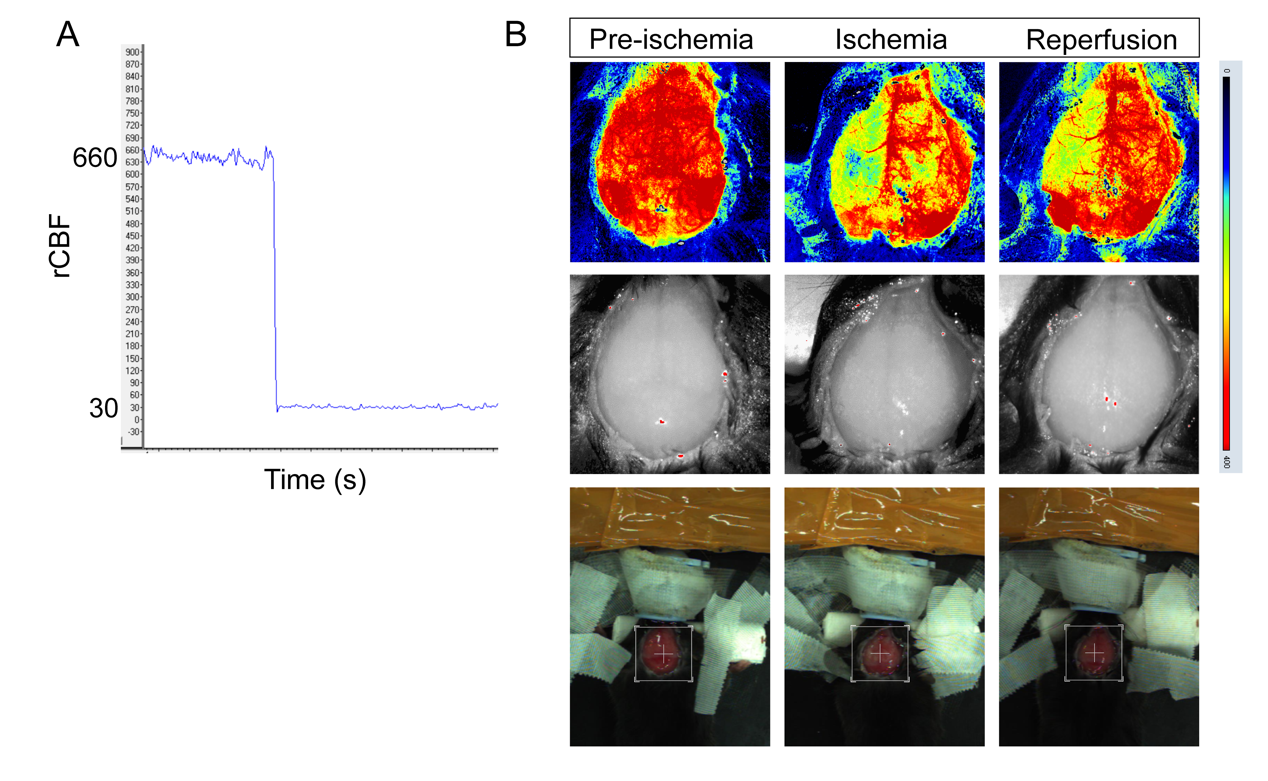
Supplementary Figure 2: Establish mice's middle cerebral artery occlusion model (MCAO). (A)** laser Doppler flowmetry was used to measure the Regional cerebral blood flow (rCBF) in all MCAO mice. **(B)** Representative photomicrographs of laser speckle imaging for MCAO mice.

**
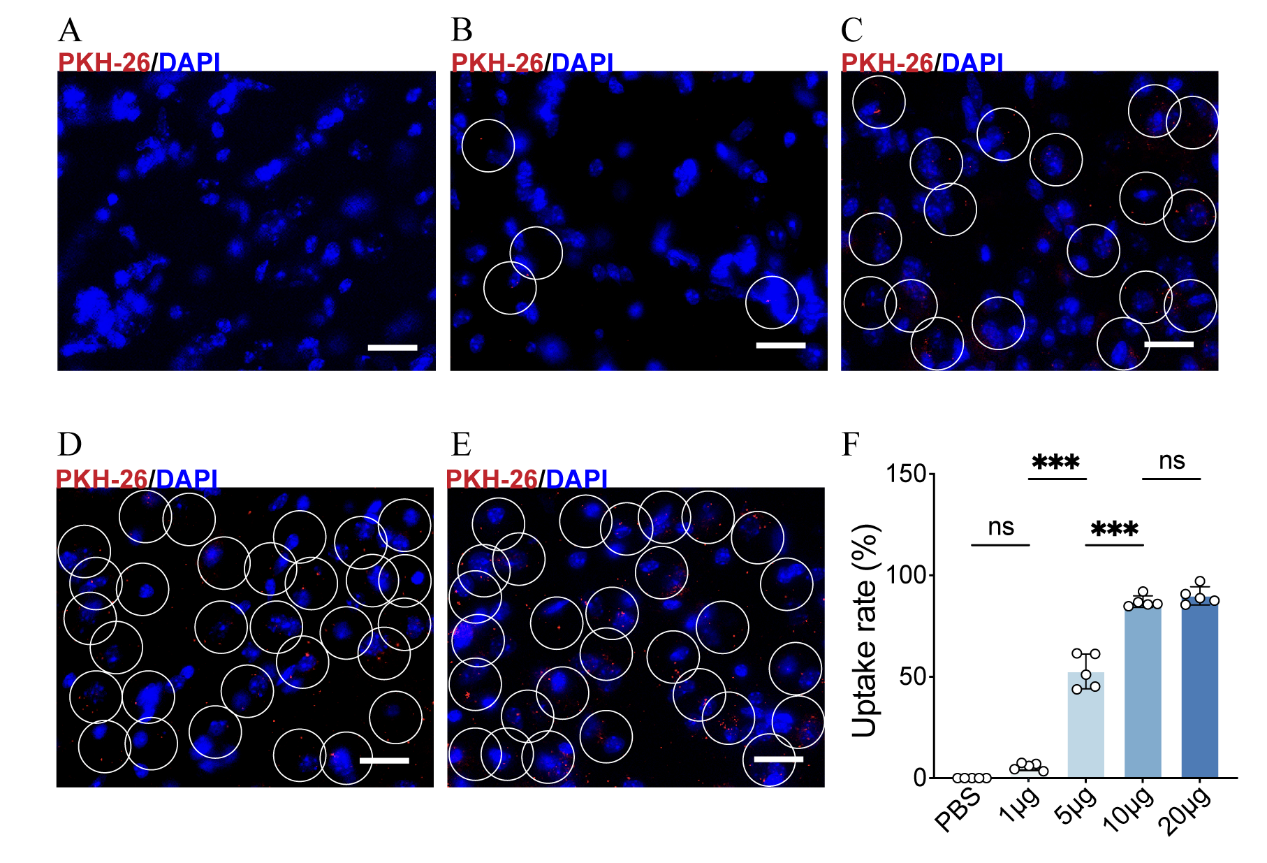
Supplementary Figure 3: The optimal dosage for intranasal administration of ADSC-Exo. (A)** The ischemic penumbra of MCAO mice was observed through fluorescence microscopy after intranasal administration of 10 μL of PBS. Scale bar = 20 μm. Following intranasal administration of 10 μL of PBS containing **(B)** 1 μg, **(C)** 5 μg, **(D)** 10 μg, or **(E)** 20 μg of ADSC-Exo, the ischemic penumbra of MCAO mice was visualized through fluorescence microscopy, showing PKH-26-labeled ADSC-Exo (red) surrounding the cell nuclei (blue). The white circles represent internalized ADSC-Exo. Scale bar = 20 μm, n = 5. **(F)** Quantitative analysis revealed the uptake rate in the ischemic penumbra after intervention with different doses of ADSC-Exo, n = 5, *** p < 0.001.


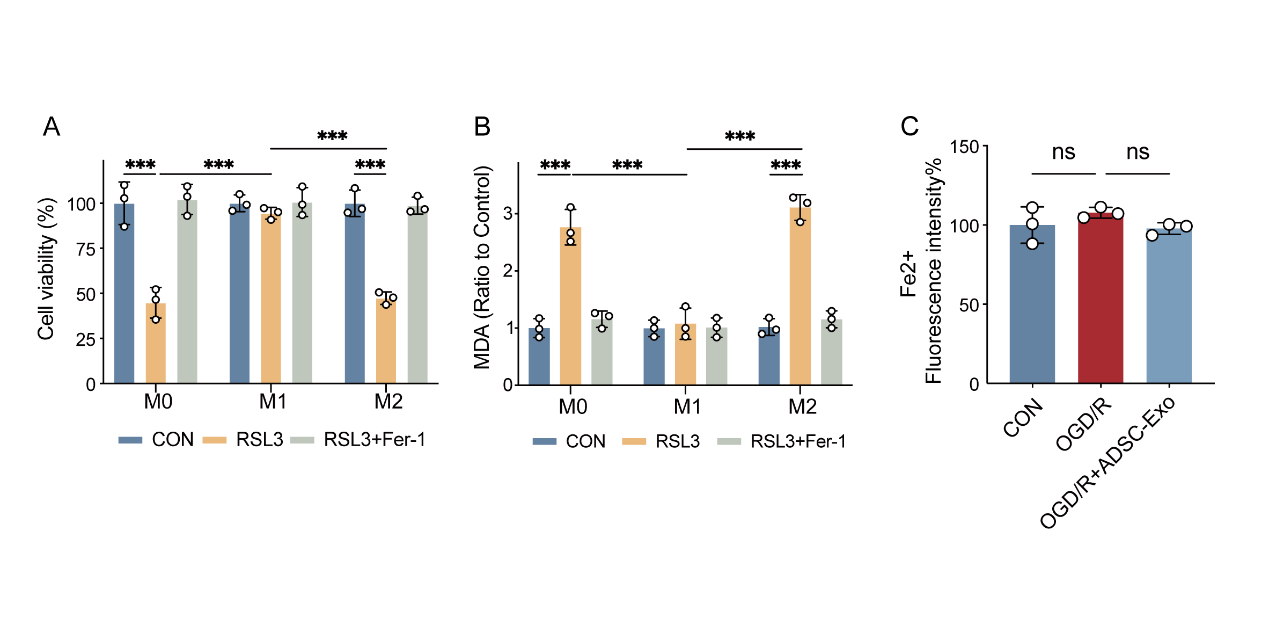
**Supplementary Figure 4:** **(A)** CCK-8 and **(B)** malondialdehyde (MDA) assays were employed to assess the sensitivity of different subtypes of microglia to ferroptosis inducer RSL-3 (500 nM, 6 h) and the rescue effect of the ferroptosis inhibitor Fer-1 (500 nM, 6 h) on different subtypes of microglia, n = 3. **(C)** The ferrous iron level (Fe2+) of M1 microglia in different groups was assessed by the FerroOrange probe, n = 3. *** p < 0.001.


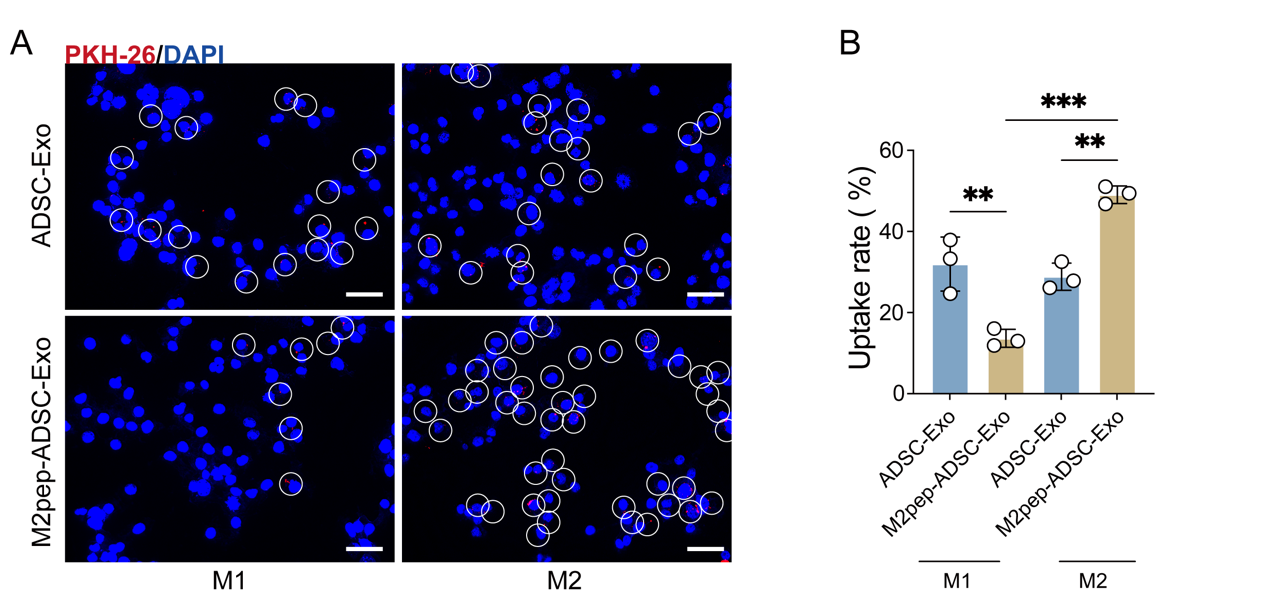


**Supplementary Figure 5: (A)** Fluorescence microscopy observation of the uptake capacity of primary microglia with different subtypes for ADSC-Exo (20 μg/mL, red) or M2pep-ADSC-Exo (20 μg/mL, red) after 1 h of co-incubation, white circles represent the internalized Exo. **(B)** Quantitative analysis of the uptake capacity of primary microglia with different subtypes for ADSC-Exo or M2pep-ADSC-Exo, n = 3.

**Supplementary Figure 6:** The re
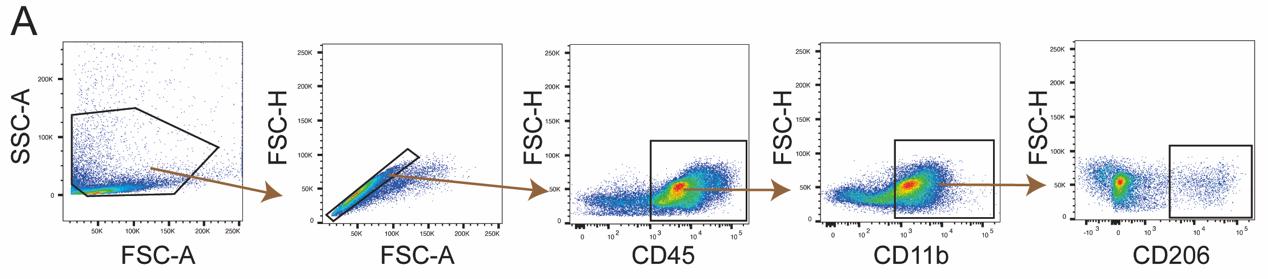
presentative flow cytometry gating plot, CD45^+^CD11b^+^ microglia, was selected, followed by further identification of CD206^+^ M2 microglia.
